# Characterizing Genes with Distinct Methylation Patterns in the Context of Protein-Protein Interaction Network: Application to Human Brain Tissues

**DOI:** 10.1371/journal.pone.0065871

**Published:** 2013-06-12

**Authors:** Yongsheng Li, Juan Xu, Hong Chen, Zheng Zhao, Shengli Li, Jing Bai, Aiwei Wu, Chunjie Jiang, Yuan Wang, Bin Su, Xia Li

**Affiliations:** College of Bioinformatics Science and Technology, Harbin Medical University, Harbin, China; University of Ulm, Germany

## Abstract

**Background:**

DNA methylation is an essential epigenetic mechanism involved in transcriptional control. However, how genes with different methylation patterns are assembled in the protein-protein interaction network (PPIN) remains a mystery.

**Results:**

In the present study, we systematically dissected the characterization of genes with different methylation patterns in the PPIN. A negative association was detected between the methylation levels in the brain tissues and topological centralities. By focusing on two classes of genes with considerably different methylation levels in the brain tissues, namely the low methylated genes (LMGs) and high methylated genes (HMGs), we found that their organizing principles in the PPIN are distinct. The LMGs tend to be the center of the PPIN, and attacking them causes a more deleterious effect on the network integrity. Furthermore, the LMGs express their functions in a modular pattern and substantial differences in functions are observed between the two types of genes. The LMGs are enriched in the basic biological functions, such as binding activity and regulation of transcription. More importantly, cancer genes, especially recessive cancer genes, essential genes, and aging-related genes were all found more often in the LMGs. Additionally, our analysis presented that the intra-classes communications are enhanced, but inter-classes communications are repressed. Finally, a functional complementation was revealed between methylation and miRNA regulation in the human genome.

**Conclusions:**

We have elucidated the assembling principles of genes with different methylation levels in the context of the PPIN, providing key insights into the complex epigenetic regulation mechanisms.

## Introduction

The postreplication addition of methyl groups to the 5-position of cytosine in certain CpG dinucleotides is one of the most widely investigated DNA modifications in mammals. Genetic studies have established that this epigenetic modification is required for embryonic development [1], genomic imprinting [2] and X-chromosome inactivation [3], and aberrant alterations in DNA methylation are linked to many human diseases, including cancers [4]. Even though the methylation level of a gene is intimately linked to its biological function, our understanding of this relationship is still coarse, fragmented and incomplete, especially in the context of protein-protein interaction network (PPIN).

Although the abundance of CpG dinucleotides in human DNA is much lower than that expected based on the GC content, the resulting dearth of CpGs is not uniformly distributed in the genome. Saxonov et al. were the firstly to investigate the genome-wide pattern of CpGs over the human genome, uncover their existence, and categorize the two classes of genes based on their CpG content [5]. Approximately 70% of human genes have high CpG content, whereas the remaining genes tend to be depleted of CpGs. Moreover, by systematically determining the methylation status of 15,609 high confidence genes, Weber et al. found that besides two distinct populations with high and low CpG frequency, a substantial overlap corresponding to genes with intermediate CpG frequency were also noted in both the populations. Based on this observation, the genes were further divided into three classes (HCPs, ICPs and LCPs) [6]. Indeed, the methylation pattern of genes in mammalian genomes exhibits substantial variations. For example, in the human genome, the CpG ratio of genes varies ∼120-fold. Recent evidences suggest a dependence of DNA methylation on local sequence content, and the CpG ratio has been widely used to measure the level of DNA methylation on an evolutionary time scale [7], [8]. These findings give us a hint that DNA methylation is primarily a function of promoter CpG content, which results in a constitutive hypo- or hypermethylated state. More recently, the generation of genome-wide DNA methylome has greatly enhanced our ability to examine the methylome and systematically dissect the methylation patterns of genes [9], [10]. Distinct patterns of methylation have been observed for genes located on different genomic regions [11]. In addition, several studies have revealed that the DNA methylation levels across samples also showed a bimodal distribution, indicating that a high proportion of genes examined were infrequently methylated, whereas a smaller proportion were highly methylated, a trend which is consistent with the results obtained in the sequence analyses [6], [9], [12]–[14]. Collectively, these findings indicate the existence of functionally relevant variations of methylation levels. By investigating the consequences of certain methylation variations, we can gain additional insights into the mechanisms and regulatory complexities of gene expression.

Recently, more and more studies are focusing on investigating the relationship between DNA methylation levels and the expression or functions of associated genes, since the seminal work by Saxonov et al. Broadly considered, house-keeping functions are significantly overrepresented in the low methylated genes (LMGs), whereas specific functions characteristic of more differentiated or highly regulated cells are significantly overrepresented in the high methylated genes (HMGs) [5]. In addition, through combined analyses of the expression levels of an extensive set of genes in 79 different tissues [15], it has been observed that genes expressed in all or almost all of the tissues are biased toward the LMG class. However, most of the previous studies have mainly focused on individual genes’ behaviours, and only limited studies have investigated the functional biases of these genes at a system level. Cellular systems use sophisticated communication between genes in order to initiate and maintain basic cellular functions such as growth, survival, proliferation and development [16], [17]. To understand the mechanisms underlying the complex biological processes, we need not only to know the components that constitute the biological system, but also the ways in which they interact with each other [18]. Protein-protein interactions are the building blocks of biological processes [19], but the roles of methylation regulation in the network remain unclear. Given that proteins are subject to variable modes of methylation regulation, we considered the assembling patterns among them in the context of the PPIN. A systematic dissection of how these genes are assembled in the network can provide new insights into the complex epigenetic regulation mechanisms underlying biological systems as well as complex phenotypes.

## Results

### Topological Features in Relation to DNA Methylation

To better understand the DNA methylation patterns in the context of PPIN, we first leveraged legacy genomic methylation profiles from four brain regions, investigating the methylome and transcriptome of 150 individuals [12]. There were 14,235 genes examined for both methylation and expression. For each gene, the level of DNA methylation is defined as a beta-value ranging from 0 to 1, with the value close to 0 indicating low levels of DNA methylation and the value close to 1 indicating high levels [20]. In total, 7891 of these genes were included in PPIN. In addition, CpG ratio is another measure of the level of DNA methylation on an evolutionary timescale, and hypermethylated genomic regions would have lower ratios, while regions that undergo low DNA methylation would maintain high ratios. Among the 14,300 genes with CpG ratios available, 7368 genes were included in the PPIN. Furthermore, in the following analyses we focused on those genes included in the PPIN, and both beta-values and CpG ratios of genes were used to further analyze the relationship between topological features and DNA methylation.

**Figure 1:**
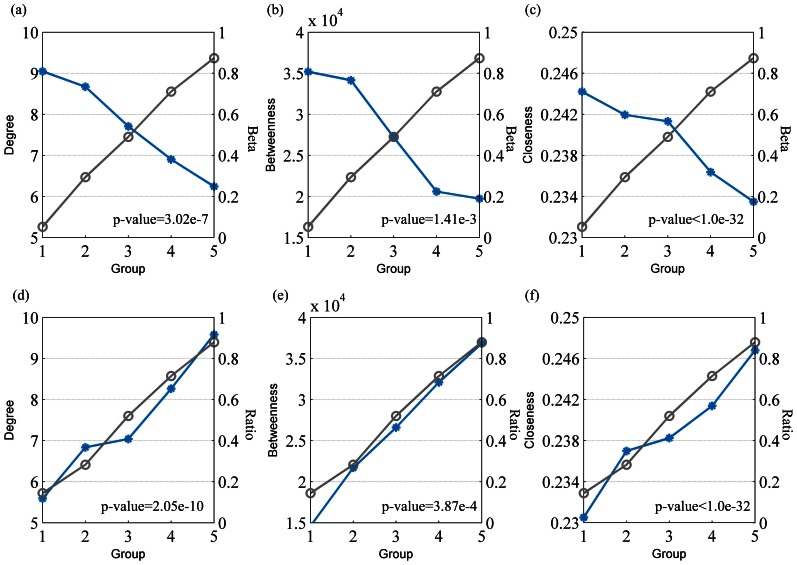
The relationship between methylation levels and topological features of genes. The genes were divided into five bins according to their methylation levels, and then the average DNA methylation levels and the average topological features were computed. The blue lines represent the distribution of topological features while the grey lines represent the average methylation levels of the genes in each bin. The genes’ methylation levels in (a)–(c) were measured by methylation microarray, while the methylation levels in (d)–(f) were measured by CpG ratios on an evolutionary timescale.

Similar to previous studies [21]–[23], the methylation levels of genes in human brain tissues also showed a bimodal distribution (Figure S1). Therefore, the genes were first classified into five bins (step by 0.2) according to their methylation levels in the brain tissues, and then the degree of differences among these gene groups was analyzed using ANOVA. As shown in Figure 1a, genes with different methylation levels (beta-values) had significant deviation in degree (*p*-value = 3.02e-7). Furthermore, the gene group with lowest methylation had the highest average degree, indicating their important roles in the PPIN. Next, we globally perturbed the methylation levels of all genes and preserved their interactions, and divided the genes into five bins as described earlier. This procedure was repeated 10,000 times. As expected, after perturbation, there was no significant change in the degree with increasing methylation levels. In addition, we also analyzed the relationships between methylation levels and another two topological features, betweenness centrality and closeness centrality. We found that with an increasing methylation level, the average betweenness and closeness centrality significantly decreased (Figure 1b and 1c, both *p*-values <0.001). Moreover, on analyzing the relationships between CpG ratio and three topological centrality measures, we obtained similar results (Figure 1d-f, *p*-values <0.001).

Based on these results, we found that there exists a potential biological association between the DNA methylation levels of genes and their topological features in the PPIN. To further analyze their assembling patterns in the PPIN, we particularly focused on two classes of genes: LMGs, consisting of genes with beta-values less than 0.2 and CpG ratio greater than 0.8, and HMGs, including genes with beta-values greater than 0.8 and CpG ratio less than 0.2. As the beta-value and CpG ratio measure the methylation levels of gene from two different aspects, this partitioning allowed us to obtain relatively more pure HMGs and LMGs and dissect more reliable assembling patterns. Finally, we allocated 1727 genes to the LMG class and 247 genes to the HMG class.

### LMGs are Centrally Located in the PPIN

**Figure 2:**
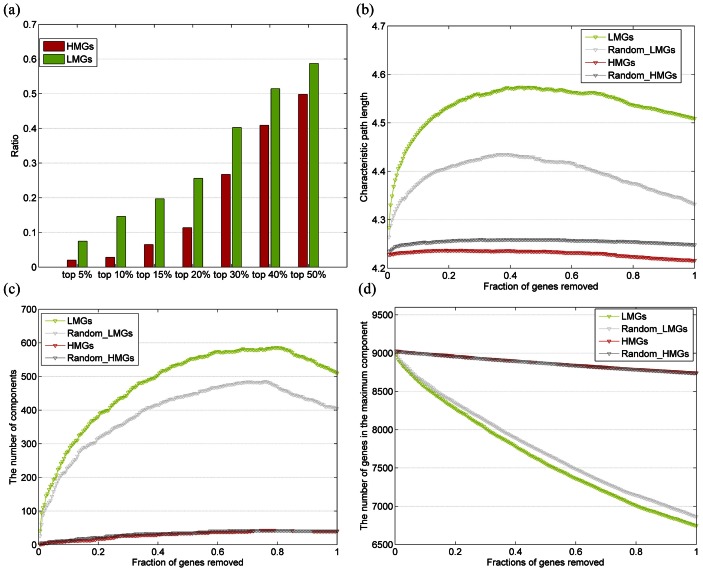
LMGs are central to network topology. (a) The percentage of LMGs and HMGs in the hubs. Genes are ranked by the degree in the PPIN and hubs are defined as the top-ranked genes. (b) The effects on the characteristic path length of the network on gradual node removal. Random removal of nodes is represented by gray lines; dark gray line represents the random removal of HMGs, while light gray line represents random removal of LMGs. Attacks against LMGs are denoted by the green line and those against HMGs are represented by the red line. (c) The number of components remaining after removing the LMGs, HMGs, and random genes. (d) The sizes of the largest remaining component after removing LMGs, HMGs and random genes.

As discussed earlier, DNA methylation levels and topological features have an inverse association. Hence, we analyzed the differences in topological features of these two gene groups. A summary of these analysis results is presented in Table 1. As shown in the table, LMGs tend to interact with more genes than HMGs and have a higher betweenness centrality. The average degree of LMGs was found to be 9.969, which is significantly higher than that of HMGs, even significantly higher than the average degree of the whole PPIN. Moreover, the average betweenness of LMGs was found to be about twice that of HMGs. These results indicate that many of the LMGs are network hubs and bottlenecks, whose values are ranked top 10% of the whole gene list; moreover, we found that the LMGs are indeed overrepresented in the top genes with high number of interactions (hubs), while the HMGs are underrepresented (Figure 2a). For example, *SMAD3*, one of the LMGs, is both a hub and bottleneck in the PPIN (degree = 178, betweenness = 1.81e6), and *SMAD* protein is well known as a signal transducer and transcriptional modulator, and functions as a transcriptional modulator activated by transforming growth factor-beta [24], [25]. Thus, it is thought to play a key role in the regulation of carcinogenesis. In the present study, we found that this gene has a low methylation level (beta-value = 0.007, R = 0.9037) and a high expression level (expression intensity values = 1024), indicating that this gene also plays important roles in the normal brain tissues.

**Table 1:** Comparisons of topological features of LMGs and HMGs.

	HPRD		LMGs		HMGs		Rank sum test
	Mean	Std	Mean	Std	Mean	Std	p-values
Degree	7.945	14.583	9.969	17.112	5.450	6.946	1.447e-5
Betweenness (*10^4^)	2.914	12.221	3.897	14.671	1.303	2.823	5.0e-8
Closeness	0.241	0.031	0.248	0.030	0.231	0.030	7.06e-15

Detection of the difference in the topological features between LMGs and HMGs could suggest a difference in the functional complexity. Both hubs and bottlenecks are known to represent important nodes in biological networks, whose changes may consequently influence a large number of interacting genes. Functional aberration of these genes may cause large-scale deleterious effects on the PPIN. Thus, we first used an *in silico* strategy that simulated the effect of specifically removing (attacking) genes in the PPIN on the characteristic path length of the main component of the network [26]. In fact, separately removal of the LMGs and HMGs from the original network has distinct effects on the overall network integrity. Moreover, successive attacks against LMGs starting from the most connected genes have a more deleterious effect on the network integrity than the removal of random proteins (Figure 2b). Conversely, removal of HMGs does not affect connectivity and thus has deleterious effects similar to the removal of random genes. The number of components and size of maximum component measure the integrity of a network from another two aspects. We found that the number of components after removal of LMGs was significantly greater than that after removal of HMGs (Figure 2c). However, the main component remaining after removing the LMGs was significantly smaller than that remaining after the removal of HMGs (Figure 2d).

Collectively, these results show that LMGs and HMGs have markedly different global properties in the PPIN. The LMGs tend to be hubs and bottlenecks in the network, indicating that they are centrally located in the PPIN, and play important roles in biological processes. In contrast, the HMGs are located in the periphery of the network. Thus, attacking LMGs may cause a more deleterious effect on the network integrity than attacking HMGs.

### Modular Organization of LMGs and HMGs in the PPIN

**Figure 3:**
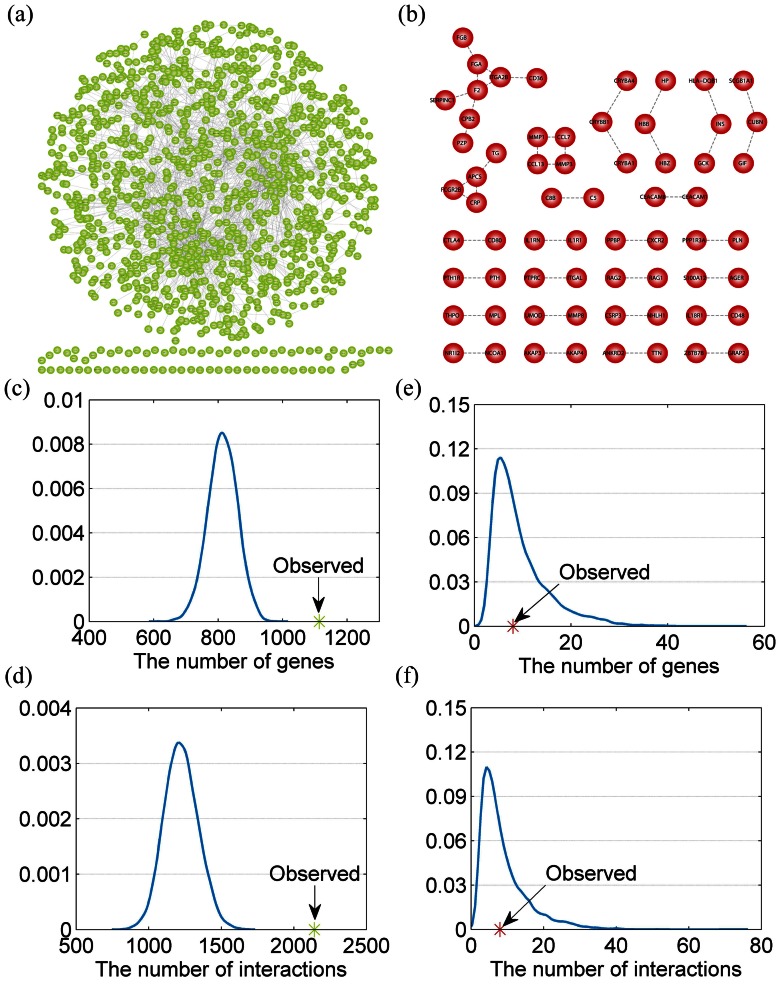
The LMG and HMG networks. (a) A PPIN connected by LMGs (LMN). (b) The protein interaction network is extracted from the PPIN which is connected by HMGs (HMN). (c) The number of vertexes of LMN is significantly larger than that of random networks (*p*-value<1.0e-4). (d) The number of edges of LMN is significantly larger than that of random networks (*p*-value<1.0e-4). (e) The number of vertexes of HMN is similar to random networks (*p*-value = 0.465). (f) The number of edges of HMN is similar to random networks (*p*-value = 0.383).

The complex functions of a living cell are carried out through the concerted activity of many genes. This activity is often coordinated by the organization of the genome into modules, which have been shown to share common functions [27]. Therefore, we analyzed the modular and community structure of these two classes of genes. After mapping these two classes of genes to the PPIN, we constructed two subnetworks of LMGs and HMGs, named as LMN and HMN, respectively (Figure 3a and 3b). As shown in Figure 3a, the maximum component of LMN consisted of 2141 interactions between 1114 genes. To evaluate whether genes in each class are significantly connected to each other, we randomly chose the same number of genes as LMGs and then obtained the maximum component of these genes. We found that none of these randomized subnetwork was denser than the real one (Figure 3c and 3d, *p*-value<1.0e-4). However, the maximum component of the HMG subnetwork only had eight genes connected by eight edges (Figure 3b), which is significantly smaller than random conditions (Figure 3e and 3f). As genes may either form certain complexes or express specific biological functions with their interacting partner genes [28], we also specified an extended subnetwork, denoted as HMN1, consisting of all HMGs and their interacting genes for further analysis. As expected, genes in the HMN1 were more densely connected than random conditions. Collectively, the above-mentioned discussion shows that the LMGs are connected to each other densely in the PPIN, indicating that they may form certain modules to express specific functions; however, the HMGs are connected with the aid of interacting partners.

**Figure 4:**
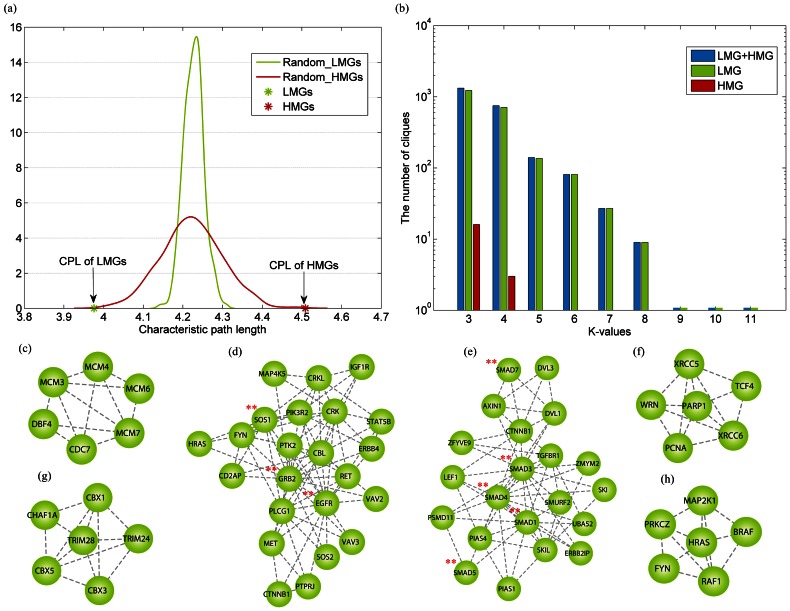
LMGs resemble functional modules in the PPIN. (a) Comparison of the average lengths of shortest paths among LMGs, HMGs, and random genes in the human protein interaction network from HPRD database. The distance between random LMGs or HMGs is fitted. (b) Number of cliques with the percentage of interesting genes greater than 0.5 at different k-values. (c)–(h) Examples of LMGs communities. Genes that have been analyzed in detail are marked with red stars.

Subsequently, we further used three common metrics to measure the modularity of a subnetwork (see Methods section), and found that the LMN showed significantly higher network modularity than that expected in random conditions (Table 2). The characteristic path lengths between the LMGs were significantly shorter (Figure 4a, 3.976 on average, *p*-value<0.001), implying that the LMGs are closer to each other. In addition, the LMN also exhibited significantly higher in-degree ratio and density. However, the characteristic path lengths between HMGs were significantly longer than random conditions (Figure 4a). The average ratio of in-degree of HMGs was only 0.032, implying that the proteins with high methylation levels may not always form a module. Conversely, the HMN1 exhibited significant modular features (Table 2). These analyses indicate that LMGs express their function in a modular pattern, and although genes with higher methylation levels might not form a network module by themselves, they are formed with the aid of their interacting partners to show significantly higher modularity.

**Table 2:** Summary of modular properties of LMGs and HMGs.

	HPRD	LMG network		H0 network		H1 network	
	Mean	Mean	p-value	Mean	p-value	Mean	p-value
In-degree ratio	N/A	0.146	<0.001	0.032	<0.001	0.261	<0.001
Density	8.77e-4	0.002	<0.001	0.001	0.046	0.007	<0.001
Characteristic path length	4.227	3.976	<0.001	4.509	1	3.769	<0.001

Additionally, to estimate if the LMGs still tend to be within the same densely connected modules detected in the original PPIN, we used the CFinder tool to identify modules from the whole PPIN. Here, a module was defined as a clique, which is a complete subgraph such that an edge is realized from every vertex to all others [29]. A module was regarded as an LMG/HMG module if more than half of its members were LMGs/HMGs. This definition reflects that the LMGs/HMGs may have dominant functions in the module. Subsequently, we counted the number of modules for the LMGs and HMGs respectively. As shown in Figure 4b, with the increase in the minimum number of genes in modules (k), a sharp decrease occurred in the number of HMG-involved modules, indicating that the HMGs do not tend to be assembled in the same modules. In contrast, the LMGs were found to participate in more modules, even some big ones. As discussed earlier, the LMGs implement functions as modules and are located in close proximity. Therefore, we carried out a detailed investigation of the modules from the LMN. In total, 327 modules were detected. As the modules were highly overlapped with each other, we further identified 13 distinct communities ranging in size from 4 to 23 when k = 4. Figure 4c–h show six communities with more than six genes. Next, we explored to observe if these communities are specifically enriched in certain cellular functions. Interestingly, the most significant relative functions of the maximum community (Figure 4d) are the functional categories related to regulation, signal transduction, and development, indicating that they play global important roles, which is in agreement with our above-mentioned analyses. In particular, the *GRB2* gene of this community was found to have 192 interacting partners in the PPIN, indicating its key role in biological processes. Evidences have shown that *GRB2* forms a complex with activated *EGFR* and the *RAS*-specific guanine nucleotide exchange factor *SOS1*, thereby mediating the growth factor-induced activation of *RAS* [30], [31]. Surprisingly, we found that these three genes are all grouped into the LMGs, implying that genes with similar functions may have similar methylation levels. Moreover, we found the second largest community that includes many members of the *SMAD* family, but none of the genes in this family were noted to be HMGs, implying that not only functionally similar genes tend to be co-methylated, genes in a family are also co-methylated with each other. Indeed, the average co-methylation levels of these two communities were found to be 0.7627 and 0.6481, respectively, which are significantly higher than random communities (*p*-values<1.0e-4); this co-methylation may imply that the genes coordinate to express their similar biological functions.

### Interaction Preferences of LMGs and HMGs

**Figure 5:**
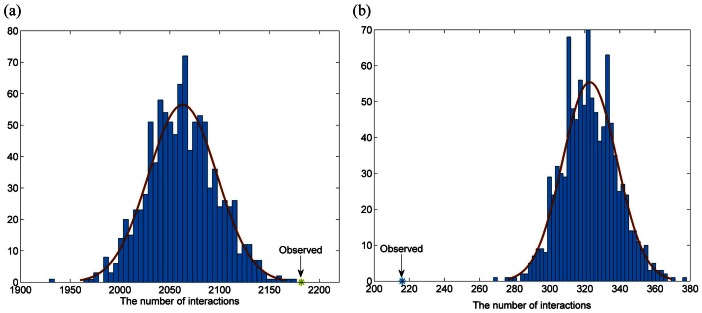
Interaction preferences of LMGs and HMGs. (a) The number of interactions within LMGs is significantly higher than that of degree-conserved random networks (*p*-value<0.001). (b) The number of interactions between LMGs and HMGs is significantly lower than that of degree-conserved random networks (*p*-value<0.001). The procedure to generate the random networks is described in Materials and Methods section.

To understand how genes with different methylation patterns assembled within the PPIN, we analyzed the interaction preferences of these two classes of genes. For this purpose, we defined interaction preference index to find out significantly over- or underrepresented interaction patterns (see details in Methods section). Consistent with our above-mentioned results, there was a significantly high density of interactions among LMGs and HMGs, implying that the intra-class communications were enhanced (Figure 5a, *p*-values<0.001). However, the interaction density between the LMGs and HMGs appeared to be extremely low. The actual number of interactions between LMGs and HMGs was 216, while in random networks these two classes of genes were averagely connected by 322.87 edges, implying that the interactions among LMGs and HMGs are significantly repressed (Figure 5b, *p*-values<0.001). According to the above-mentioned discussions and special interaction preferences between and within the two classes of genes, we obtained the communication patterns between the genes with different methylation levels in the context of PPIN. As the LMGs are centrally located in the PPIN, they tend to express their biological functions in a modular pattern. On the other hand, as the HMGs are located in the periphery of the PPIN, they have relatively lower modularity. The communications between intra-class genes were both enhanced; however, the LMGs, with the help of other genes (e.g., intermediate methylated genes (IMGs)), were found to communicate with the HMGs. These observations suggest that genes with similar methylation levels tend to interact with each other, while those with considerable difference in the methylation levels tend to avoid interactions. Indeed, in one of our recent studies, we found that physically interacting gene pairs have a similar methylated pattern; i.e., they have higher co-methylation level than random pairs. Therefore, systematic dissection of the assembling pattern of genes with differential methylation levels may provide new insights into the complex regulatory mechanisms.

### Differences in Expression and Functions between the LMGs and HMGs

**Figure 6:**
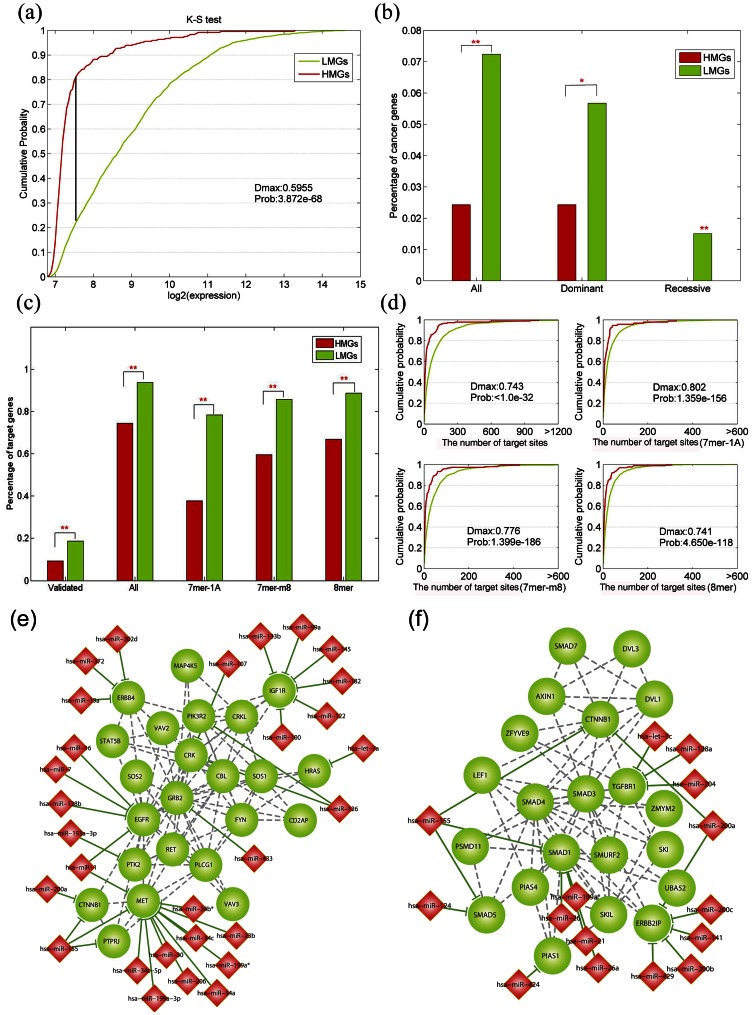
LMGs and HMGs are significantly different in expression pattern, functions and miRNA regulations. (a) The cumulative distribution functions of gene expression for LMGs (green) and HMGs (red). (b) Comparison of the percentage of cancer genes. Cancer genes are further divided into dominant and recessive cancer genes according to the annotations of cancer gene census. (c) Comparison of the percentage of miRNA targets. The experimentally validated target genes have been retrieved from four manually curated databases, while the predicted miRNA targets have been collected from TargetScan and further divided into three types of targets. (d) The cumulative distribution functions of the number of miRNA target sites in LMGs (green) or HMGs (red). The maximum distance between these two distributions and the probabilities are computed by the Kolmogorov-Smirnov (K-S) test. (e) and (f) Two LMG communities that are regulated by miRNAs. The red diamonds represent miRNAs, while the green circles represent LMGs (The red stars above the bars indicate the significant levels, **p<0.01, *p<0.05).

It has been reported that DNA methylation has different propensities to regulation messages in different biological processes or functional categories. For example, in animals, genes expressed in more tissues are biased to be low methylated while genes that are expressed in only a small number of tissues tend to be high methylated [5]. Indeed, we found that the expression patterns between LMGs and HMGs are significantly different (*p*-value = 3.87e-68, Kolmogorov-Smirnov test), and that the most of the LMGs are highly expressed genes (Figure 6a), implying their key roles in brain tissues. Subsequently, we explored if these two groups of genes express different functions in biological systems. According to the above-mentioned analyses, the LMGs tend to be hubs and bottlenecks in the PPIN, playing important roles in maintaining the stability of the network. As expected, the LMGs are overrepresented in the basic biological functions, such as binding activity and regulation of transcription, while the HMGs are overrepresented in sophisticated functions categories, such as “chemotaxis,” “inflammatory response,” and “immune response” (Table 3).

**Table 3:** Distributions of top 15 GO terms for the LMG and the HMG classes.

GO code	GO term description	Appearances		P-value
		LMGs	HMGs	
**Over-represented in class LMG**				
GO:0005634	(CC) nucleus	0.511	0.190	<1.0e-32
GO:0005737	(CC) cytoplasm	0.479	0.247	7.26e-12
GO:0005515	(MF) protein binding	0.524	0.304	9.20e-11
GO:0005654	(CC) nucleoplasm	0.131	0.036	1.67e-5
GO:0005730	(CC) nucleolus	0.155	0.053	1.74e-5
GO:0000166	(MF) nucleotide binding	0.200	0.089	2.67e-5
GO:0003677	(MF) DNA binding	0.145	0.049	2.85e-5
GO:0005829	(CC) cytosol	0.228	0.113	3.86e-5
GO:0006355	(BP) regulation of transcription, DNA-dependent	0.130	0.049	2.37e-4
GO:0005524	(MF) ATP binding	0.146	0.073	1.78e-3
GO:0010467	(BP) gene expression	0.077	0.024	2.45e-3
GO:0006915	(BP) apoptotic process	0.071	0.020	2.53e-3
GO:0003700	(MF) sequence-specific DNA binding transcription factor activity	0.102	0.045	3.98e-3
GO:0000122	(BP) negative regulation of transcription from RNA polymerase II promoter	0.064	0.024	1.28e-2
GO:0045892	(BP) negative regulation of transcription, DNA-dependent	0.054	0.020	2.30e-2
**Over-represented in class HMG**				
GO:0006935	(BP) chemotaxis	0.003	0.061	<1.0e-32
GO:0009897	(CC) external side of plasma membrane	0.005	0.069	<1.0e-32
GO:0006954	(BP) inflammatory response	0.011	0.109	<1.0e-32
GO:0006955	(BP) immune response	0.011	0.130	<1.0e-32
GO:0005615	(CC) extracellular space	0.031	0.247	<1.0e-32
GO:0005576	(CC) extracellular region	0.054	0.360	<1.0e-32
GO:0007267	(BP) cell-cell signaling	0.013	0.093	7.55e-15
GO:0005887	(CC) integral to plasma membrane	0.042	0.146	1.74e-11
GO:0007601	(BP) visual perception	0.005	0.040	2.94e-8
GO:0007166	(BP) cell surface receptor signaling pathway	0.010	0.057	3.06e-8
GO:0004872	(MF) receptor activity	0.058	0.146	3.42e-7
GO:0008544	(BP) epidermis development	0.003	0.032	4.08e-7
GO:0005102	(MF) receptor binding	0.012	0.057	7.08e-7
GO:0005529	(MF) sugar binding	0.005	0.036	1.37e-6
GO:0004888	(MF) transmembrane signaling receptor activity	0.003	0.028	1.49e-6

P values were calculated by using the chi-square statistic. Only top 15 terms significant at the 0.05 level are presented. Parenthesized markings stand for the three major sub-ontologies comprising GO: CC for “cellular component,” BP for “biological process,” and MF for “molecular function”.

In addition, several genes are known to be a typical class of genes that play specific roles in cellular systems; it is interesting to examine the DNA methylation pattern of these genes, which may provide new insights into understanding the mechanism of complex diseases. Cancer is a common complex disease, and many genes have been reported as involved in the development of cancer. Aberrant methylation of CpG islands in promoter regions is known to be involved in multiple types of cancers. First, we explored the methylation patterns of known cancer genes. As expected, we found that cancer genes are significantly over-represented in the LMGs (Figure 6b, *p*-value = 0.0045, Fisher’s exact test), indicating that cancer genes tend to have low methylation levels, which is consistent with a recent study [12]. Hypermethylation is one of the major epigenetic modifications that repress transcription via promoter regions of tumor suppressor genes [32], [33]. We subsequently compared the DNA methylation patterns in two major classes of cancer genes: dominant and recessive cancer genes [34]. After excluding four genes with ambiguous classification in the database, among the 470 cancer genes, there were 365 dominant cancer genes and 105 recessive cancer genes. Interestingly, the dominant cancer genes were found to be slightly overrepresented in the LMG class (*p*-value = 0.0327, Fisher’s exact test), but no recessive cancer gene was observed in the HMG class, indicating that recessive genes tend to avoid methylation in normal tissues. Biologically, this finding is reasonable. As evidences have shown that recessive genes are frequently hypermethylated in cancers, their low methylation level in normal states promises a wider range to change in cancers. Furthermore, through selective aberrant methylation of the LMGs, which are hubs and bottleneck proteins, cancer may regulate the PPIN in a wider scope, and attacking these genes has more deleterious effects on the stability of cellular systems.

Essential genes are another class of functionally important genes, which have been comprehensively analyzed previously. When we compared the distribution of essential genes in the two classes of genes with different methylation levels, we found that essential genes were significantly overrepresented in the LMGs than HMGs, indicating that essential genes also tend to have low methylation levels in normal tissues (*p*-value = 0.0068, Fisher’s exact test). Aging is another complex process in addition to genetic diseases controlled by both genetic and epigenetic factors. As aging is one of the important factors to induce diseases, investigation of the methylation pattern of aging genes is helpful to understand the nature of diseases [14], [35], [36]. As expected, the aging genes were also found to be over-represented in the LMG class (*p*-value = 0.0316, Fisher’s exact test), indicating that aging genes tend to have lower methylation levels. Previous studies have shown that aging genes have higher degree and betweenness in the PPIN, which is in accordance with the characteristic of the LMG class [37].

### Functional Complementation between Methylation and microRNA Regulation

DNA methylation in the 5′-promoter regions of the genes and miRNA regulation at the 3′-untranslated regions are two major epigenetic regulation mechanisms. Both these regulations can affect the gene expression and their corresponding protein products. However, limited studies have investigated the relationship between DNA methylation and miRNA regulation [8]. Unexpectedly, when comparing the miRNA regulation patterns of LMGs and HMGs, we found that LMGs tend to be regulated by miRNAs (Figure 6c, *p*-values<0.001, Fisher’s exact test). About 93.69% of LMGs were predicted to be target genes of miRNAs, which is about 1.3-fold higher than that of HMGs (*p*-value<1.0e-32, Fisher’s exact test). We found that the trend is clearer in the “experiment validated target set” than in the “predicted target set” (the ratio was about twice the value, *p*-value = 3.03e-4, Fisher’s exact test). Most importantly, when focusing on the miRNA key targets which were manually retrieved from more than 3,000 literatures [38], 30% of these key targets were observed to be LMGs, whereas only 1.64% of the key targets were represented in the HMG class. Moreover, in the LMG class, it was found that the genes’ 3′-UTR tend to include more miRNA target sites than the HMGs, implying higher miRNA regulatory complexity of these genes (Figure 6d, *p*-value<1.0e-32, Kolmogorov-Smirnov test). In other words, the genes under stronger promoter DNA methylation control tend to avoid miRNA regulation, and vice versa, indicating functional complementation between transcriptional methylation regulation and posttranscriptional miRNA regulation in the human genome.

### Robustness of the Organizational Principles of Genes with Distinct Methylation Patterns

The use of large-scale protein-protein interaction and DNA methylation data as the basis of the present study proved to be useful for gaining biological knowledge. Although these datasets are far from being complete and may contain some noises, it is unlikely that the incompleteness or noise can totally distort the obtained results. All the information in Human Protein Reference Database (HPRD) had been manually extracted from the literature by expert biologists who read, interpret and analyze the published data. Thus, the overall signal inferred from our analyses is strong enough to reflect real biology. In addition, we also analyzed the organizational patterns in the context of another two PPINs-one being the integrated PPIN from a total of 21 different databases [39] and the other being predicted based on protein structures [40], and found that the results obtained in our analyses are robust (see detailed results in Text S1).

Similar to previous studies on other tissues, the current study revealed that the genes’ methylation level in the human brain tissues also follows a bimodal distribution. In the above-mentioned analyses, to obtain more pure LMGs and HMGs, we had used the commonly employed threshold (0.2/0.8) to define the two classes of genes, which may bring about some false negatives. By relaxing the definitions of LMGs and HMGs, we also used another threshold (0.3/0.7) that had been employed in some studies to define the LMGs and HMGs [41], [42]. As a result, we found most of the results remaining stable (see details in Text S2), further mirroring the actual assembling patterns of genes with different methylation levels in the context of the PPIN.

## Discussion

Our study revealed a negative correlation between topological centralities and DNA methylation regulations in the brain tissues. That is, for proteins centrally located in the PPIN, the corresponding genes avoid to be regulated by DNA methylation. By focusing on two main classes of genes with considerably different methylation levels in the brain tissues, we elucidated the assembling principles of genes with different methylation levels in the context of PPIN. The LMGs are centrally located in the PPIN and have higher expression levels than the HMGs, and express their functions in a modular pattern. In contrast, the HMGs are located in the periphery of the PPIN and form functional modules with the aid of their interacting partners. Furthermore, genes with similar methylation levels tend to interact with each other, while those with considerable difference in methylation levels tend to avoid interactions.

By further analyzing the function and expression of these two classes of genes, it was found that most of the LMGs are highly expressed genes and tend to be functionally important genes, such as cancer genes, essential genes, and aging genes. Many previous exploratory studies have been carried out to understand how DNA methylation and miRNA regulate the expression of their target genes; however, most of them have focused on the effect of each mechanism on the expression of target genes. Most interestingly, we found that the LMGs tend to be further regulated by miRNAs, implying functional complementation between transcriptional methylation regulation and posttranscriptional miRNA regulation in the human genome. As shown in Figure 6e and 6f, some LMGs in these two communities were observed to be under strict miRNA regulations. For example, the *MET* gene was found to be regulated by 11 miRNAs in the experiment validated target set and has been shown to be the key target of four miRNAs (hsa-miR-1, hsa-miR-206, hsa-miR-23b and hsa-miR-34a). In the brain, the *MET* gene is expressed in developing circuits that are involved in social behavior and communication. Disturbances in *MET* expression may contribute to a syndrome that includes autism and co-occurring gastrointestinal dysfunction [43]. As hsa-miR-1 is a brain and muscle-tissue-specific miRNA [44], these strict regulations of *MET* would reasonably be more beneficial in stabilizing its expression in brain tissue. Another example is the *EGFR* gene, an interacting partner of *MET*, which is regulated by four miRNAs. Evidences have revealed that the activation of *EGFR* stimulates multiple pathways of signal transduction, leading to a wide range of cellular responses. Aberrant overexpression of *EGFR* has been observed in glioma [45]. The above-mentioned studies suggest that although the *EGFR* plays important roles in the development of brain, its abnormal overexpression could result in the progression of glioma. Additionally, the *EGFR* promoter is hypermethylated in both low-and high-grade glioblastoma. The hsa-miR-7, which targets *EGFR*, is a potential tumor suppressor in glioblastoma targeting critical cancer signalling pathways. Kefas et al. have identified miR-7 downregulation in glioblastoma tissue, when compared with the adjacent brain. They noted that the miR-7 directly targets *EGFR*, thus decreasing its level in glioblastoma cells [46]. Furthermore, it has been demonstrated that transfection of miR-7 reduced the viability and invasiveness of glioblastoma cells. In another gene community, it has been observed that some genes with important roles are also tightly regulated by miRNAs (Figure 6f), such as *SMAD1*, *ERBB2IP*, and *TGFBR1*. We propose that this sophisticated regulation of gene expression may be achieved by the functional complement of methylation and miRNA regulations, and abnormal regulations may result in complex phenotypes. First, the low methylation levels of a gene promise a high expression potentiality, while the strict miRNA regulations avoid its aberrant high expression. Through extension, if a gene is under stronger promoter DNA methylation control, then its expression will become low enough and may not need miRNA to further repress its expression. In contrast, if the DNA methylation regulation of a gene is weak, then the cellular system may adopt miRNA’s fine-tune regulation to avoid its aberrant high expression in the cells. Systematic analysis of multiple layers of regulation may provide new insights into the nature of human diseases. In addition, LMGs and HMGs are over- and underrepresented in hubs, respectively, suggesting that DNA methylation selectively avoids targeting the network hubs, promising their high expression adequate to play their important functions. Meanwhile, miRNAs target the network hubs in a fine-tune pattern to avoid their aberrant high expression. By selectively regulating the network hubs, which in turn affects a larger amount of neighboring cellular components, DNA methylation can regulate cellular networks on a larger scale.

Moreover, we found that the genes in functional modules or the same gene family tend to be under similar DNA methylation regulations; i.e., they are co-methylated with each other, which is in agreement with the previous findings that genes encoding interacting proteins tend to have similar mRNA expression profiles. Analyzing DNA methylation regulation in the context of the PPIN provides important insights into how the dynamics of a biological system can be efficiently controlled.

By considering other independent PPINs obtained by different technologies, the main results obtained in the present study are robust. Although it has been reported that there might be weak experimental bias with respect to abundant proteins showing more PPI partners in yeast [47], the identified correlation between degree and abundance depends on the technology to detect protein-protein interactions. However, there is no correlation for abundance-independent technology (such as structure-based or yeast two-hybrid). In the structure-based PPIN used in the present study, this association was not observed (R = −0.0162, P = 0.0875), suggesting that the differences between LMGs and HMGs are robust to PPI detection bias.

Finally, we noted that the present study may significantly contribute to both the PPIN and DNA methylation research: On the one hand, by incorporating DNA methylation data, our results make important steps to reveal the dynamic properties and organizational principles of genes with different methylation levels in the context of the human PPIN. On the other hand, this study also highlights the potential to improve current aberrant methylated genes identification by adding protein-protein interaction data. However, all the results analyzed in this study are based on methylation dataset from normal brain tissues; we presumed that this analysis strategy can be extended to other tissues with datasets having more samples. In addition, in the current study, we only focused on the LMGs and HMGs, and it might be interesting to determine where the intermediate class belongs. On comparing the topological features of the IMGs with LMGs, HMGs, and the whole PPIN, we found that the IMGs have the intermediate degree, betweenness and closeness, similar to randomly selected genes (see details in Text S3). In summary, our study provides insights into the assembling patterns of genes with different methylation levels, and the implication of this study will impact the understanding of normal cellular functions and the mechanisms of tumorigenesis and aging process.

### Conclusions

Our results reveal the organization principles of genes with different methylation levels in the context of PPIN. Importantly, we found that LMGs in normal brains play central roles in the network, whose functional aberrances have deleterious effects on the biological system. In addition, together with the recent findings, we observed that the LMGs tend to be regulated by miRNAs, implying functional complementation between transcriptional methylation regulation and posttranscriptional miRNA regulation in the human genome. Analysis of DNA methylation regulation in the context of the PPIN not only provides important insights into how the dynamics of a biological system can be efficiently controlled, but also has implications for the functional interpretation of mechanisms underlying complex phenotypes.

## Materials and Methods

### The Human PPIN

The human protein-protein interaction data were downloaded from HPRD (release 9) [47]. To measure the DNA methylation levels of the transcripts, we retrieved Entrez gene IDs for all the transcripts that were listed in the HPRD interactome with RefSeq identifiers. After removing entries that lacked Entrez gene identifiers and only considering the maximum component of the interactome, the PPIN that we analyzed contained 35,865 interactions among 9028 proteins. In addition, another two human PPINs were considered – one is integrated interactions from 21 different databases and the other is predicted based on protein structures. For the integrated PPIN, each interaction was required to be supported by at least one piece of direct experimental evidence demonstrating physical association between these two human proteins [39]. After mapping these proteins to Entrez genes, there were 76,049 interactions among 9692 genes in the maximum component. The structure-based prediction of protein-protein interactions was kindly provided by Zhang et al [40], which contains 371,741 interactions between 14,091 genes in the maximum component.

### Analysis of DNA Methylation and Gene Expression Data

In our study, two types of datasets were used to measure the DNA methylation levels of protein-coding genes: DNA methylomes of human brain tissues and CpG ratios at the DNA sequence level. The former dataset reported by Gibbs JR et al. investigated the DNA methylomes of four human brain regions each from 150 individuals (600 samples in total) [12]. In total, the DNA methylation levels of 27,578 CpG sites spanning more than 14,000 genes were measured by the Illumina Infinium HM27 DNA assay, and the beta-values were used to quantitatively measure the DNA methylation levels of specific CpG sites, ranging from 0 (completely unmethylated) to 1 (completely methylated) [48]. On the other hand, we used CpG ratios of high- confidence promoters to represent the DNA methylation patterns in the human genome [11]. The GC content and the ratio of the observed vs. expected CpG dinucleotides in sliding 500-bp windows with 5-bp offset were first determined, and then the CpG ratio was calculated using the following formula: (number of CpGs×number of bp)/(number of Cs×number of Gs). After mapping to Entrez gene identifiers, 14,300 promoters were subsequently analyzed.

The matched mRNA expression dataset was also collected from Gibbs JR et al, and raw intensity values for each probe were first transformed using the rank invariant normalization method and then using log2 method. In our analysis, only samples with both DNA methylation and gene expression datasets were used, which included 475 samples of four human brain regions.

### Compilation of Gene Sets with Specific Functions

The aging genes were downloaded from the GenAge database, which collected human aging-related genes after an extensive review of the literatures [49]. In total, we retrieved 261 human aging genes for further analysis. In addition, we obtained 474 human cancer genes and their corresponding annotation information from the Cancer Gene Census database [34]. These cancer genes could be further divided into two major types of cancer genes: dominant cancer genes and recessive cancer genes. After excluding four genes with ambiguous classification in the database, among the 470 cancer genes, there were 365 dominant cancer genes and 105 recessive cancer genes. The third kind of important genes that we considered was essential genes, and human essential genes were obtained from MGD [50], [51]. For the phenotype data, lethality postnatal (MP: 0005373) and lethality-prenatal/perinatal (MP:0005374) were treated as lethal phenotypes. Finally, 2667 mouse-lethal human orthologs were collected as human essential genes.

### Data of miRNA Targets

Two sets of miRNA target genes were used in our analyses. First, a set of experimentally validated target genes were extracted from TarBase [52], miRTarBase [53], miRecords [54], and miR2Disease [55], assembling 3131 regulations among 311 miRNAs and 1761 genes, and defined as “experiment validated target set”. In addition, another set of conserved miRNA targets predicted by TargetScan [56] were downloaded and there were a total of 15,031 targets for all human miRNAs, and were defined as “predicted target set”. As TargetScan predicts biological targets of miRNA by searching for the presence of conserved 8mer and 7mer sites that match the seed region of each miRNA, the targets were further divided into three types: an exact match to positions 2–8 of the mature miRNA followed by an “A” (8mer), an exact match to positions 2–8 of the mature miRNA (7mer-m8), and an exact match to positions 2–7 of the mature miRNA (the seed) followed by an “A” (7mer-1A).

### Topological Features in the PPIN

Topological centrality in the PPIN can be characterized by three widely used topological features: degree, betweenness centrality and closeness centrality. Degree is defined as the number of connections a gene has with other genes. Betweenness centrality, which represents how influential a gene is in communicating between node pairs, is defined by 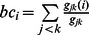, where 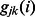 is the number of shortest path between node *j* and *k* across gene *i*, and 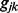 is the total number of shortest paths connecting nodes *j* and *k*. Closeness centrality is defined as the mean shortest path between a gene and all other genes reachable from it. The greater these measures of a node are, the more central it is. In addition, hub and bottleneck genes are defined as the percent of genes that are top-ranked (5–50%), according to degree and betweenness. Moreover, we also used the number of network components and the size of maximum component that remains after gradual removal of genes to evaluate the centrality of a gene set.

### Modularity

To investigate whether gene subnetworks form any modules, we used the term “in-degree” of a gene to represent the number of its within-subnetwork connections, and “out-degree” for its outside-subnetwork connections. The in-degree ratio is used to measure the modularity of a gene set, which is defined as the ratio of in-degree. Moreover, we also used the density and average characteristic path length to measure the modularity of the gene sets. Characteristic path length is the average of shortest paths between the nodes. Density is defined as the ratio between the number of total edges in a subnetwork and the total number of possible edges.

### Interaction Preference

To evaluate how closely two classes of genes or genes belonging to the same class interact with each other, we introduced a concept of interaction preference index. Interaction preference index quantitatively assesses the extent of interactions of genes between two classes or within the same class in the actual protein network, when compared with random cases. Here, random cases mean the average number of interactions of the considered genes in 1000 degree preserving random networks. And the interaction preference index is significantly higher than 1 indicates that the interactions are enhanced. In contrast, the interactions are suppressed if this measure is significantly lower than 1.

### Overrepresented Functional Categories of a Gene Set

The functional annotation of genes was obtained from the NCBI gene database, and then the chi-square test was used to identify the significantly overrepresented functional categories of a specific gene set. Functional categories with an adjusted *p*-value less than 0.05 and annotated by at least five genes were considered in our analyses.

### Randomization Test

To evaluate whether the genes in one class are significantly connected with each other, we computed the number of nodes and edges in the actual networks, and then randomly selected the same number of genes in the PPIN and computed these two measures. This process was repeated 10,000 times. The *p*-value is the fraction of the nodes (or edges) in random cases, which is greater than that in the real condition. We also used the same approach to determine the significance of the modularity measures of the LMGs and HMGs.

The statistical significance of the interaction preference of a gene set or between two gene sets is determined by the fraction of values in random networks, which is higher (or lower) than the actual ones. In the present study, 1000 random networks that have the same degree of distribution as that of the original network were generated.

## Supporting Information

Figure S1 **Density plots of gene DNA methylation from human brain tissues.**
(DOC)

Text S1 **Robustness of the organizational principles of genes with distinct methylation patterns in the context of another two PPINs.**
(DOC)

Text S2 **The organizational principles of genes with distinct methylation patterns are robust to different thresholds.**
(DOC)

Text S3 **The topological features of intermediate methylated genes.**
(DOC)
