# Supplementary material for: Characterizing Genes with Distinct Methylation Patterns in the Context of Protein-Protein Interaction Network: Application to Human Brain Tissues
Source: PLoS One. 2013 Jun 12;8(6):e65871. doi: 10.1371/journal.pone.0065871 (PMC3680465; doi:10.1371/journal.pone.0065871)
Supplement: Figure S1 — Density plots of gene DNA methylation from human brain tissues. (DOC) [file pone.0065871.s001.doc]

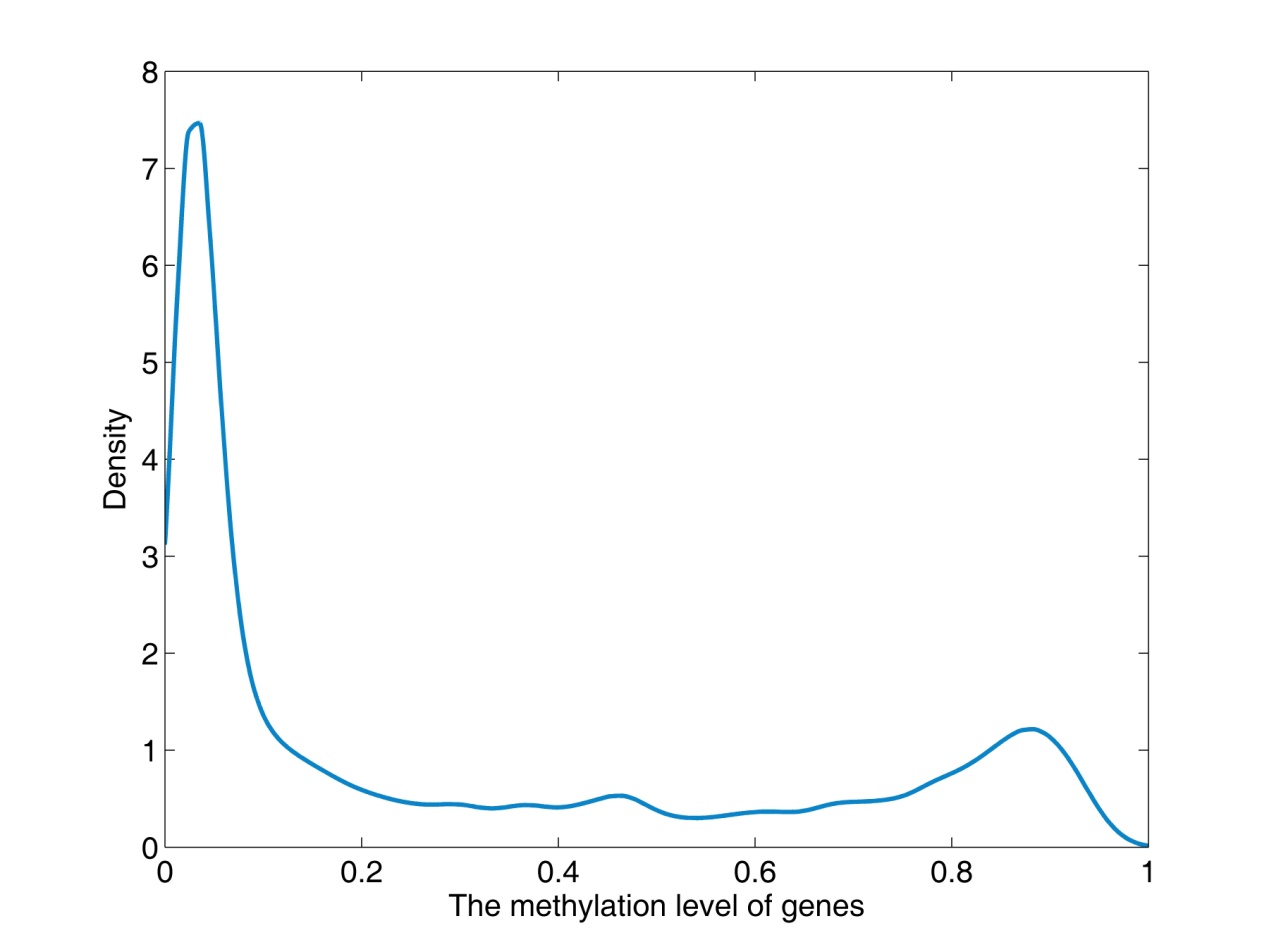


**Figure S1. Density plots of gene DNA methylation from human brain tissues.** The DNA methylation level of genes exhibits distinctive ‘‘bimodal’’ patterns previously observed.
