# Supplementary material for: Characterizing Genes with Distinct Methylation Patterns in the Context of Protein-Protein Interaction Network: Application to Human Brain Tissues
Source: PLoS One. 2013 Jun 12;8(6):e65871. doi: 10.1371/journal.pone.0065871 (PMC3680465; doi:10.1371/journal.pone.0065871)
Supplement: Text S1 — Robustness of the organizational principles of genes with distinct methylation patterns in the context of another two PPINs. (DOC) [file pone.0065871.s002.doc]

**Text S1. Robustness of the organizational principles of genes with distinct methylation patterns in the context of another two protein-protein interaction networks**

To test if the results obtained in the text are bias to the protein interaction network we used, we explored the assembling patterns of genes with distinct methylation patterns in another two protein-protein interaction networks (PPINs).

1. The organizational principles of genes with distinct methylation patterns in the context of integrated PPIN (Page 2-7)
2. The organizational principles of genes with distinct methylation patterns in the context of structure-based PPIN (Page 8-12)

**I. The organizational principles of genes with distinct methylation patterns in the context of integrated PPIN**

To construct a global human physical protein interaction network, Ben Lehner et al. integrated data from 21 different sources to define a network of 80,922 physical interactions that can occur among 10,229 human proteins . These interactions are supported by at least one piece of direct experimental evidence demonstrating physical association between two human proteins. After mapping these proteins to Entrez genes, there are 76,049 interactions among 9,692 genes in the maximum component. Using the 0.2/0.8 as threshold, finally, we allocated 1,902 genes to the LMG class and 282 genes to the HMG class.

**LMGs are located in the central of protein interaction network**

A summary of the analysis results is listed in Table S1. Our analysis shows that LMGs tend to interact with more genes than HMGs and have a higher betweenness centrality. The average degree of LMGs is 21.187, which is significantly higher than that of HMGs, even the average degree of the whole PPIN. Moreover, the average betweenness of LMGs is about three times to that of HMGs.

**Table S1. Comparisons of topological features of LMGs and HMGs**.

|  | IPPIN | | LMGs | | HMGs | | Rank sum test |
| --- | --- | --- | --- | --- | --- | --- | --- |
|  | Mean | Std | Mean | Std | Mean | Std | p-values |
| Degree | 15.693 | 31.559 | 21.187 | 37.914 | 5.560 | 7.386 | 2.535e-21 |
| Betweenness (*10^4^) | 2.852 | 10.848 | 3.938 | 12.675 | 1.210 | 2.502 | 2.192e-9 |
| Closeness | 0.258 | 0.033 | 0.262 | 0.033 | 0.236 | 0.029 | 6.700e-34 |

Moreover, we found the LMGs are indeed overrepresented in the top genes with high number of interactions (hubs) but the HMGs are underrepresented (Figure S1a). Attacking LMGs may cause a more deleterious effect on the network integrity than that of HMGs (Figure S1b-d).


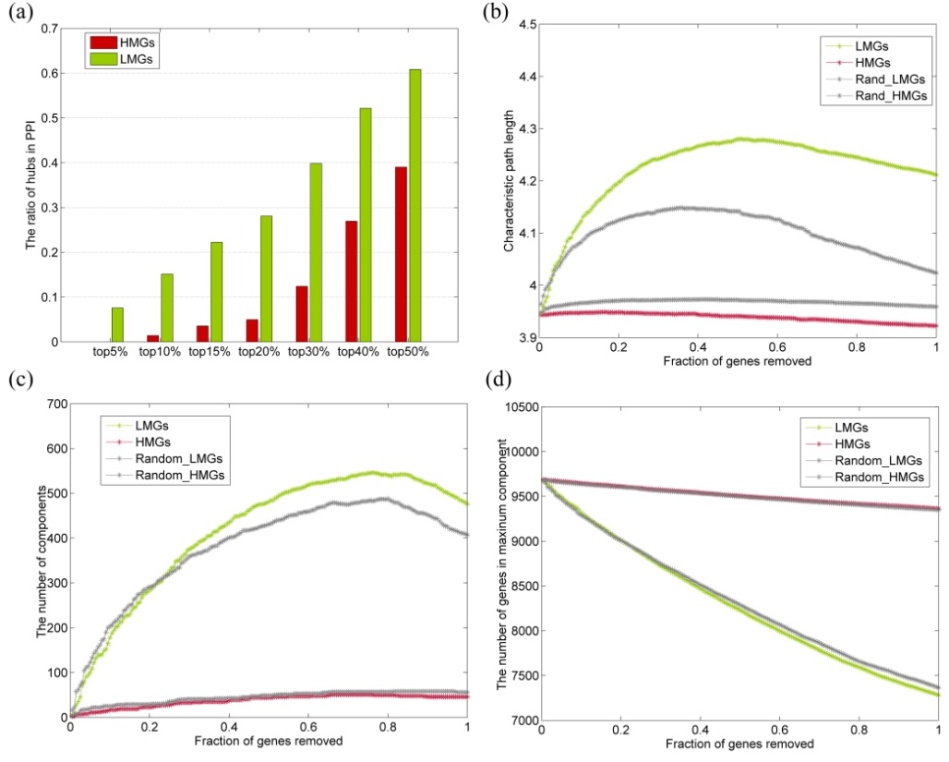


**Figure S1. LMGs are central to network topology.** (a) The percentage of LMGs and HMGs in the hubs. Genes are ranked by the degree in the PPIN and hubs are defined as the top ranked genes. (b) The effects on the characteristic path length of the network on gradual node removal. Random removal of nodes is represented by the grey lines, dark grey represents the random removal of HMGs while light grey line represents random removal of LMGs, attacks against LMGs by the green line, attacks against HMGs by the red line. (c) The number of components remaining after removing the LMGs, HMGs and random genes. (d) The sizes of the largest remaining component after removing LMGs, HMGs and random genes.

**Modular organization of LMGs and HMGs in the protein interaction network**

We analyzed the modular and community structure of these two classes of genes. After mapping these two classes of genes to the protein interaction network, we constructed two networks of LMGs and HMGs, named as LMN and HMN. The maximum component of LMN consists of 1,389 genes, and it is much larger than expected by chance (Figure S2a, p-value<1.0e-4). Furthermore, there are 5,491 edges between genes in the maximum component, which is much denser than expected by chance (Figure S2c, p-value<1.0e-4). However, the maximum component of HMG network only has ten genes connected by 12 edges, which is significantly smaller than random conditions (Figure S2b, d). We also specified an extended subnetwork for further analysis, denoted as HMN1, consisting of all the interacting genes of HMGs. As expected, genes in the HMN1 are more densely connected than random conditions.

In order to further explore the significance of modular composition of the LMN and HMN, and see if it is possible to achieve similar level of dense connections in random conditions, we used three metrics to measure the modularity of a network (see methods). As a result, we found the LMN shows significantly higher network modularity than what would be expected in random genes (Table S2).

**Table S2. Summary of modular properties of LMGs and HMGs**.

|  | IPPIN | LMG network | | H0 network | | H1 network | |
| --- | --- | --- | --- | --- | --- | --- | --- |
|  | Mean | Mean | p-value | Mean | p-value | Mean | p-value |
| In-degree ratio | N/A | 0.159 | <0.001 | 0.033 | 0 | 0.215 | <0.001 |
| Density | 1.6E-3 | 0.003 | <0.001 | 0.001 | 0.9000 | 0.007 | <0.001 |
| Characteristic path length | 3.9432 | 3.662 | <0.001 | 4.414 | 1 | 3.622 | <0.001 |


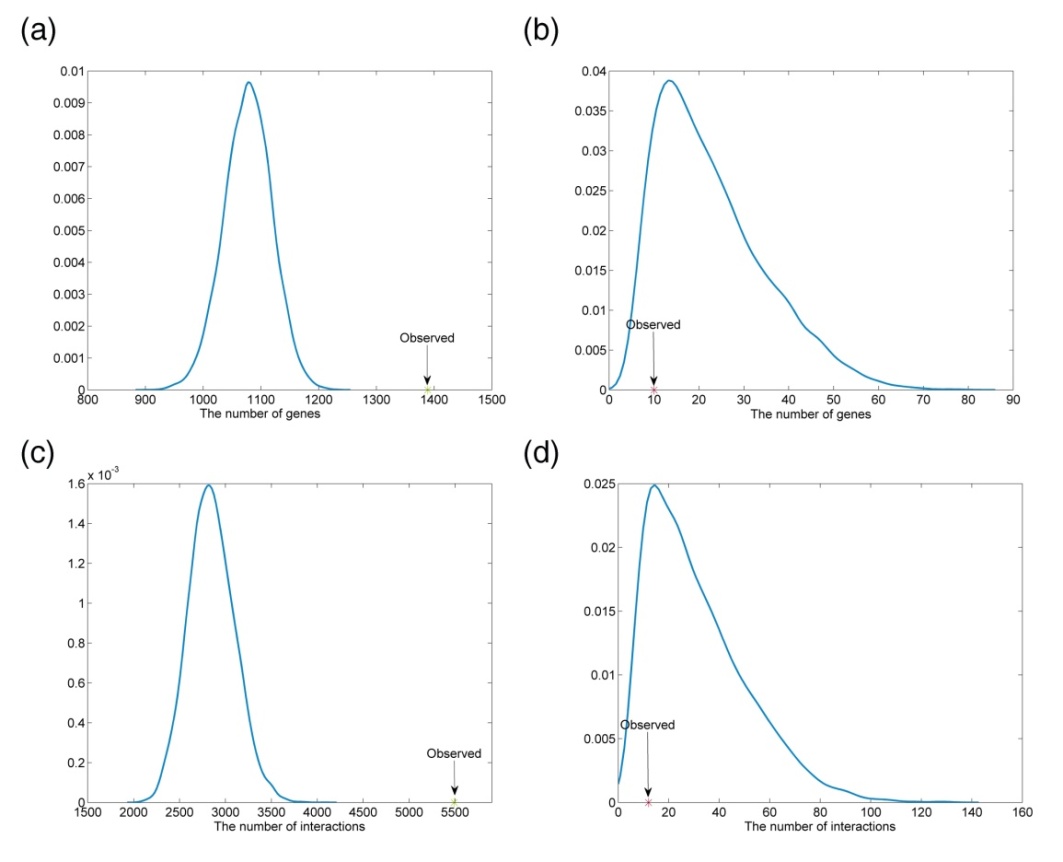


**Figure S2. The LMG and HMG networks.** (a) The number of vertexes of LMN is significantly larger than that of random networks. (b) The number of vertexes of HMN is similar to random networks. (c) The number of edges of LMN is significantly larger than that of random networks. (d) The number of edges of HMN is similar to random networks.

**Interaction preferences of LMGs and HMGs**

In order to understand how genes with different methylation levels assembled within the protein interaction network, here, we analyzed the interaction preferences of these two classes of genes. For this purpose, we defined interaction preference index to find out significant over-represented or under-represented interaction patterns (see details in methods). Consistent with our results in main text, there is a significantly high density of interactions among LMGs or among the HMGs, implying the communications intra-class are enhanced (Figure S3a, p-values<0.001). However, the interaction density between LMGs and HMGs appears to be extremely low, and the interactions among LMGs and HMGs are significantly repressed (Figure S3b, p-values<0.001).


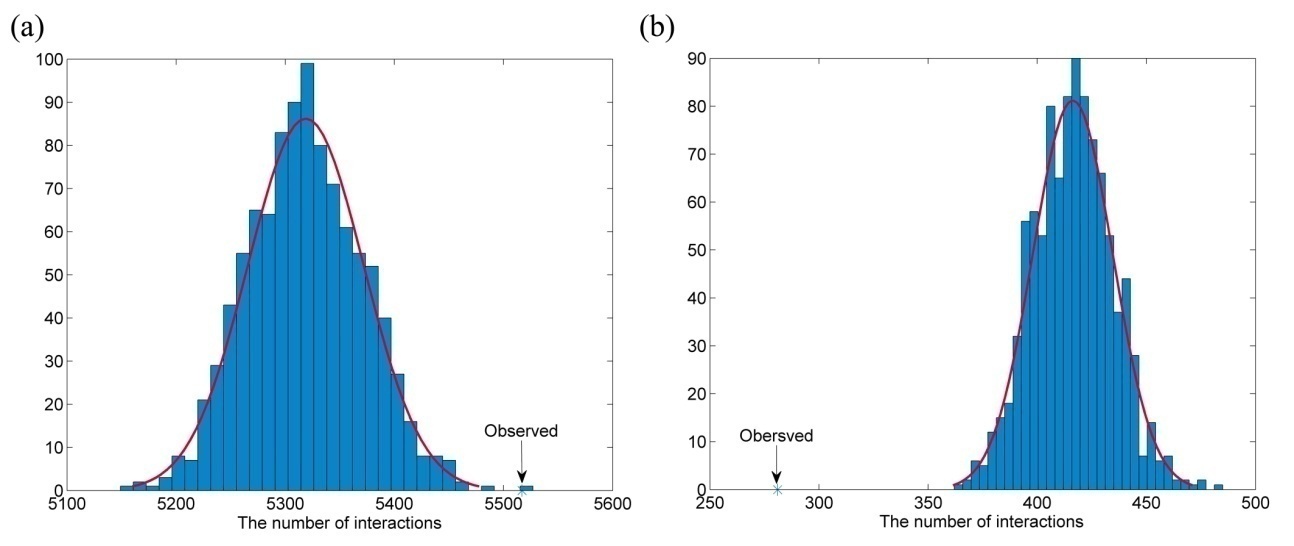


**Figure S3. Interaction preferences of LMGs and HMGs.** (a) The number of interactions within LMGs is significantly larger than that of degree-conserved random networks (p-value<0.001). (b) The number of interactions between LMGs and HMGs is significantly smaller than that of degree-conserved random networks (p-value<0.001). The procedure to generate the random networks is described in materials and methods.

**Differences in expression and functions between the LMGs and HMGs**

We found the expression patterns between LMGs and HMGs are significantly different (p-value=4.246e-74, Kolmogorov-Smirnov test), and the LMGs are enriched in high expression genes (Figure S4a), implying its key roles in brain tissues. In addition, some genes may belong to a class of genes that play specific roles in cellular systems; it is interesting to examine the DNA methylation pattern of these genes, which may provide new insight into understanding the mechanism of complex diseases. Firstly, we explored the methylation patterns of cancer genes. As expected, we found the cancer genes are significantly over-represented in the LMGs (Figure S4b, p-value=0.0083, Fisher’s exact test), indicating cancer genes tend to have low methylation levels, which is consistent with a recent study. We next compared the DNA methylation patterns between two major classes of cancer genes: dominant and recessive cancer genes. After excluding four genes with ambiguous classification in the database, among the 470 cancer genes, there were 365 dominant cancer genes and 105 recessive cancer genes. Interestingly, the dominant cancer genes are slightly overrepresented in the LMG class (p-value=0.0579, Fisher’s exact test), while recessive cancer genes are slightly over-presented in the HMG class, indicating that recessive genes tend to avoid methylation in normal tissues. When we compared the distribution of essential genes in two classes of genes with different methylation levels, we found essential genes are significantly overrepresented in LMGs than HMGs, indicating essential genes also tend to have low methylation levels in normal tissues (p-value=0.0018, Fisher’s exact test). Finally, we found the aging genes are also over-represented in the LMG class (p-value=0.0091, Fisher’s exact test), indicating aging genes tend to have lower methylation levels.


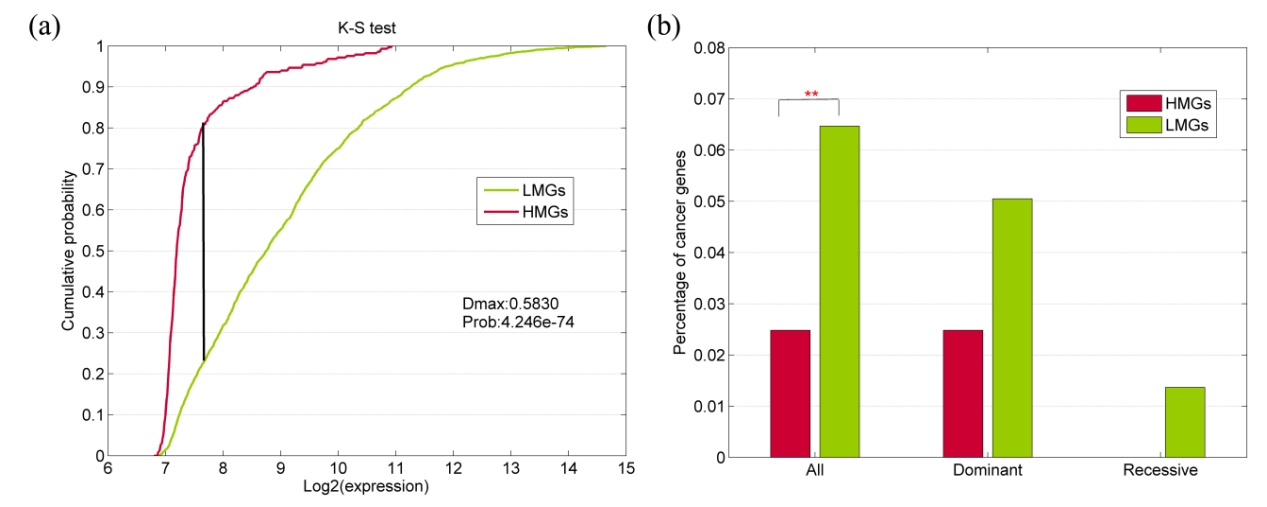


**Figure S4. LMGs and HMGs are significantly different in expression pattern and functions.** (a) The cumulative distribution functions of gene expression for LMGs (green) and HMGs (red). (b) Comparison of the percentage of cancer genes. Cancer genes are further divided into dominant and recessive cancer genes according the annotations of cancer gene census.

**Functional complementation between methylation and microRNA regulation**

Unexpectedly, when comparing the miRNA regulation pattern of LMGs and HMGs, we found LMGs tend to be regulated by miRNAs (Figure S5a, p-values<0.001, Fisher’s exact test). About 92.95% of LMGs are predicted to be miRNA target gene set, which is about 1.22 fold to that of HMGs (p-value<1.0e-32, Fisher’s exact test). We found the trend is clearer in the “experiment validated target set” than in the “predicted target set” (the ratio is about 2.2 times, p-value=3.676e-5, Fisher’s exact test), suggesting that the false positive information incorporated in the “predicted target set” leads to a lower fold. Most importantly, when focusing on the miRNA key targets, we found 34.25% of these key targets are LMGs whereas only 1.71% key targets are represented in the HMG class. Moreover, the genes in LMG class regulated by miRNAs tend to have more miRNA regulations than HMGs, implying more miRNA regulatory complexity of these genes (Figure S5b, p-value=2.577e-22, Kolmogorov-Smirnov test).


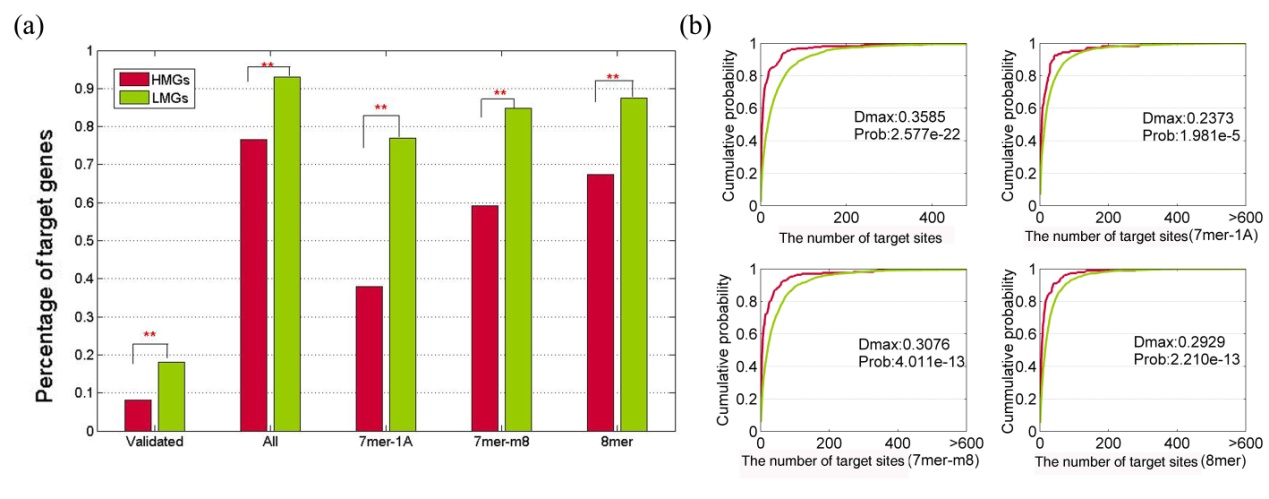


**Figure S5. LMGs and HMGs are significantly different in miRNA regulations.** (a) Comparison of the percentage of miRNA targets. The experimental validated target genes are retrieved from four manually curated databases while the predicted miRNA targets are collected from TargetScan and further divided into three types of targets. (b) The cumulative distribution functions of the number of miRNAs that regulate LMGs (green) or HMGs (red). The maximum distance between these two distributions and the probabilities are computed by the Kolmogorov-Smirnov (K-S) test.

**II. The organizational principles of genes with distinct methylation patterns in the context of structure-based PPIN**

Recently, Zhang et al. have provided an integrated resource that combines the predicted and experimentally determined protein-protein interactions using a Bayesian framework . We also analyzed the assembling patterns of genes with distinct methylation patterns in brain tissues in the context of the integrated PPIN, which includes 371,741 interactions between 14,091 genes in the maximum component. Among the 14,235 genes with DNA methylation in the microarray, 11,078 genes are included in the IPPIN; and among the 14,300 genes with CpG ratios, 10,784 genes are included in the IPPIN. As the beta values and CpG ratios measure the methylation levels of genes from two different aspects, finally, we allocated 2,294 genes to the LMG class and 409 genes to the HMG class using the 0.2/0.8 as threshold.

## LMGs are located in the central of protein interaction network

A summary of the analysis results is listed in Table S3. Our analysis shows that LMGs tend to interact with more genes than HMGs and have a higher betweenness centrality. The average degree of LMGs is 64.370, which is significantly higher than that of HMGs, even the average degree of the whole PPIN. Moreover, the average betweenness of LMGs is about two times to that of HMGs.

**Table S3. Comparisons of topological features of LMGs and HMGs.**

|  | IPPI | | LMGs | | HMGs | | Rank sum test |
| --- | --- | --- | --- | --- | --- | --- | --- |
|  | Mean | Std | Mean | Std | Mean | Std | p-values |
| Degree | 52.763 | 84.699 | 64.370 | 100.010 | 44.443 | 73.00 | 0.0039 |
| Betweenness (*10^4^) | 3.357 | 13.402 | 5.077 | 17.203 | 2.249 | 9.258 | 2.450e-9 |
| Closeness | 0.3019 | 0.0421 | 0.3116 | 0.0425 | 0.2953 | 0.0412 | 3.389e-12 |

Moreover, we found the LMGs are indeed overrepresented in the top genes with high number of interactions (hubs) but the HMGs are underrepresented (Figure S6a). Attacking LMGs may cause a more deleterious effect on the network integrity than that of HMGs (Figure S6b-d).


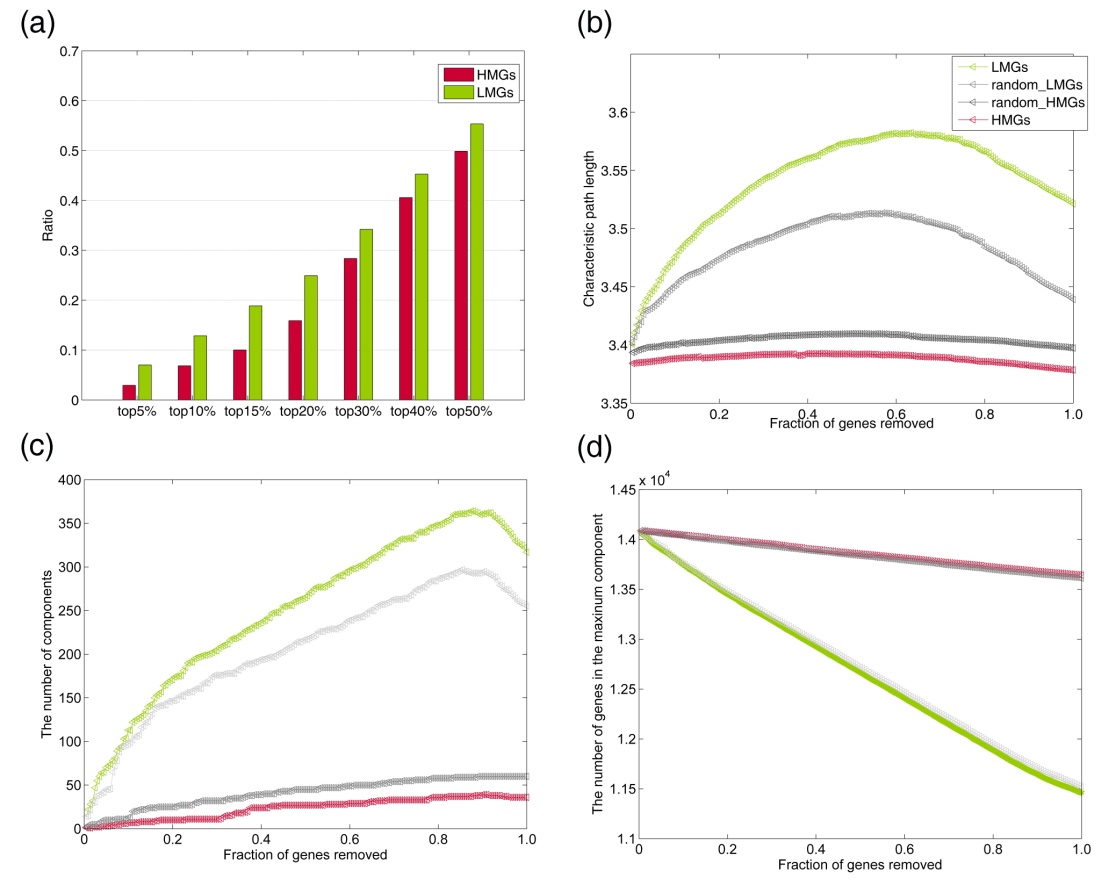


**Figure S6. LMGs are central to network topology.** (a) The percentage of LMGs and HMGs in the hubs. Genes are ranked by the degree in the PPIN and hubs are defined as the top ranked genes. (b) The effects on the characteristic path length of the network on gradual node removal. Random removal of nodes is represented by the grey lines, dark grey represents the random removal of HMGs while light grey line represents random removal of LMGs, attacks against LMGs by the green line, attacks against HMGs by the red line. (c) The number of components remaining after removing the LMGs, HMGs and random genes. (d) The sizes of the largest remaining component after removing LMGs, HMGs and random genes.

**Modular organization of LMGs and HMGs in the protein interaction network**

We analyzed the modular and community structure of these two classes of genes. After mapping these two classes of genes to the protein interaction network, we constructed two networks of LMGs and HMGs, named as LMN and HMN. The maximum component of LMN consists of 1,888 genes, and it is much larger than expected by chance (Figure S7a, p-value<1.0e-4). Furthermore, there are 18,093 edges between genes in the maximum component, which is much denser than expected by chance (Figure S7c, p-value<1.0e-4). However, the maximum component of HMG network only has ten genes connected by 12 edges (Figure S7b), which is significantly smaller than random conditions (Figure S7b, d). We also specified an extended subnetwork for further analysis, denoted as HMN1, consisting of all the interacting genes of HMGs. As expected, genes in the HMN1 are more densely connected than random conditions.


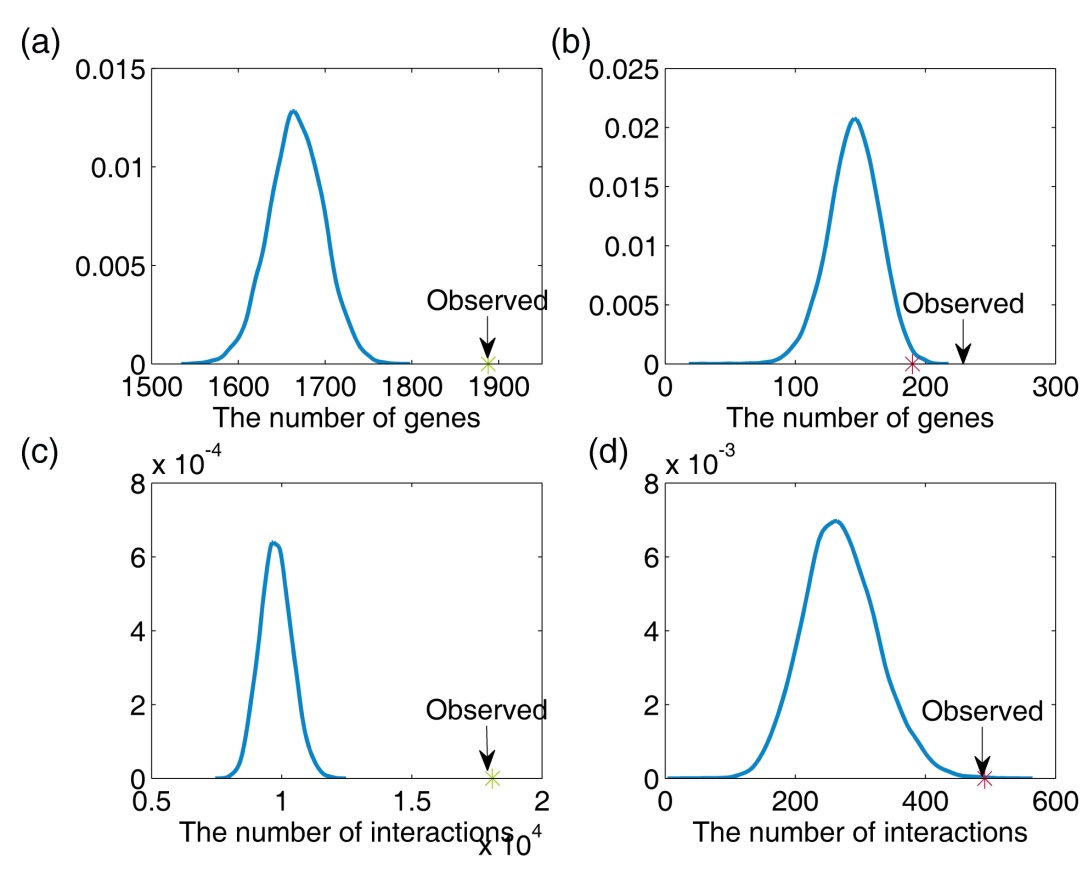


**Figure S7. The LMG and HMG networks.** (a) The number of vertexes of LMN is significantly larger than that of random networks (p-value<1.0e-4). (b) The number of vertexes of HMN is similar to random networks. (c) The number of edges of LMN is significantly larger than that of random networks (p-value<1.0e-4). (d) The number of edges of HMN is similar to random networks.

In order to further explore the significance of modular composition of the LMN and HMN, and see if it is possible to achieve similar level of dense connections in random conditions, we used three metrics to measure the modularity of a network (see methods). As a result, we found the LMN shows significantly higher network modularity than what would be expected in random genes (Table S4).

**Table S4. Summary of modular properties of LMGs and HMGs**.

|  | IPPIN | LMG network | | H0 network | | H1 network | |
| --- | --- | --- | --- | --- | --- | --- | --- |
|  | Mean | Mean | p-value | Mean | p-value | Mean | p-value |
| In-degree ratio | N/A | 0.140 | <0.001 | 0.031 | <0.001 | 0.756 | <0.001 |
| Density | 0.0037 | 0.007 | <0.001 | 0.006 | <0.001 | 0.017 | <0.001 |
| Characteristic path length | 3.3821 | 3.148 | <0.001 | 3.452 | 0.8950 | 2.819 | <0.001 |

**Interaction preferences of LMGs and HMGs**

In order to understand how genes with different methylation levels assembled within the protein interaction network, here, we analyzed the interaction preferences of these two classes of genes. For this purpose, we defined interaction preference index to find out significant over-represented or under-represented interaction patterns (see details in methods). Consistent with our results in main text, there is a significantly high density of interactions among LMGs or among the HMGs, implying the communications intra-class are enhanced (Figure S8a, p-values<0.001). However, the interaction density between LMGs and HMGs appears to be extremely low, and the interactions among LMGs and HMGs are significantly repressed (Figure S8b, p-values<0.001).


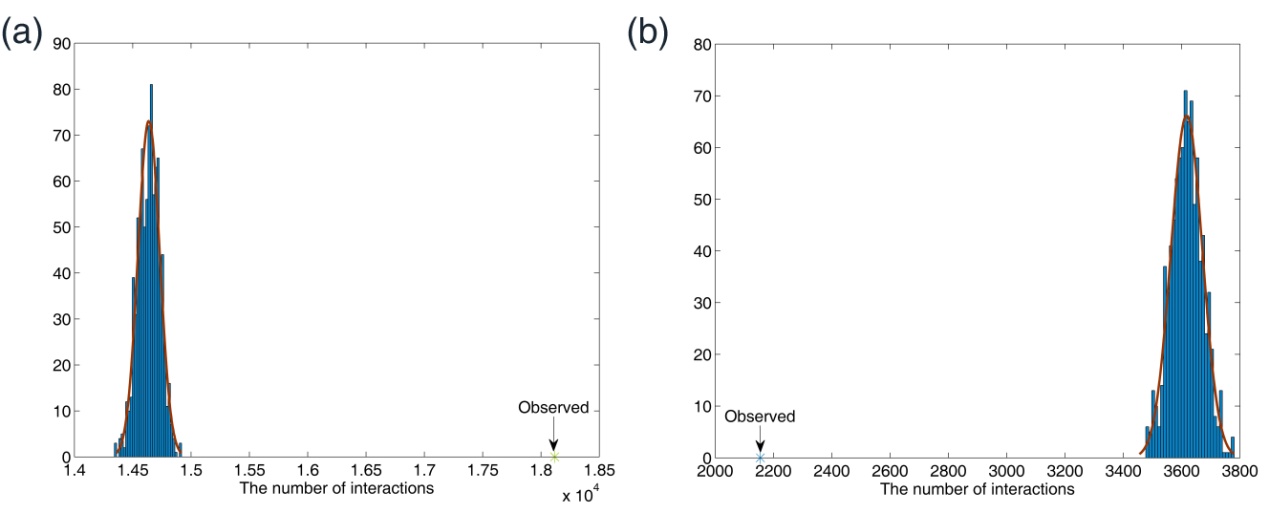


**Figure S8. Interaction preferences of LMGs and HMGs.** (a) The number of interactions within LMGs is significantly larger than that of degree-conserved random networks (p-value<0.001). (b) The number of interactions between LMGs and HMGs is significantly smaller than that of degree-conserved random networks (p-value<0.001). The procedure to generate the random networks is described in materials and methods.

**Differences in expression and functions between the LMGs and HMGs**

We found the expression patterns between LMGs and HMGs are significantly different (p-value=7.021e-161, Kolmogorov-Smirnov test), and the LMGs are enriched in high expression genes (Figure S9a), implying its key roles in brain tissues. In addition, some genes may belong to a class of genes that play specific roles in cellular systems; it is interesting to examine the DNA methylation pattern of these genes, which may provide new insight into understanding the mechanism of complex diseases. Firstly, we explored the methylation patterns of cancer genes. As expected, we found the cancer genes are significantly over-represented in the LMGs (Figure S9b, p-value=1.23e-4, Fisher’s exact test), indicating cancer genes tend to have low methylation levels, which is consistent with a recent study. We next compared the DNA methylation patterns between two major classes of cancer genes: dominant and recessive cancer genes. After excluding four genes with ambiguous classification in the database, among the 470 cancer genes, there were 365 dominant cancer genes and 105 recessive cancer genes. Interestingly, the dominant cancer genes are slightly overrepresented in the LMG class (p-value=0.0026, Fisher’s exact test), while recessive cancer genes are slightly over-presented in the HMG class, indicating that recessive genes tend to avoid methylation in normal tissues. When we compared the distribution of essential genes in two classes of genes with different methylation levels, we found essential genes are significantly overrepresented in LMGs than HMGs, indicating essential genes also tend to have low methylation levels in normal tissues (p-value=5.81e-10, Fisher’s exact test). Finally, we found the aging genes are also over-represented in the LMG class (p-value=0.0047, Fisher’s exact test), indicating aging genes tend to have lower methylation levels.


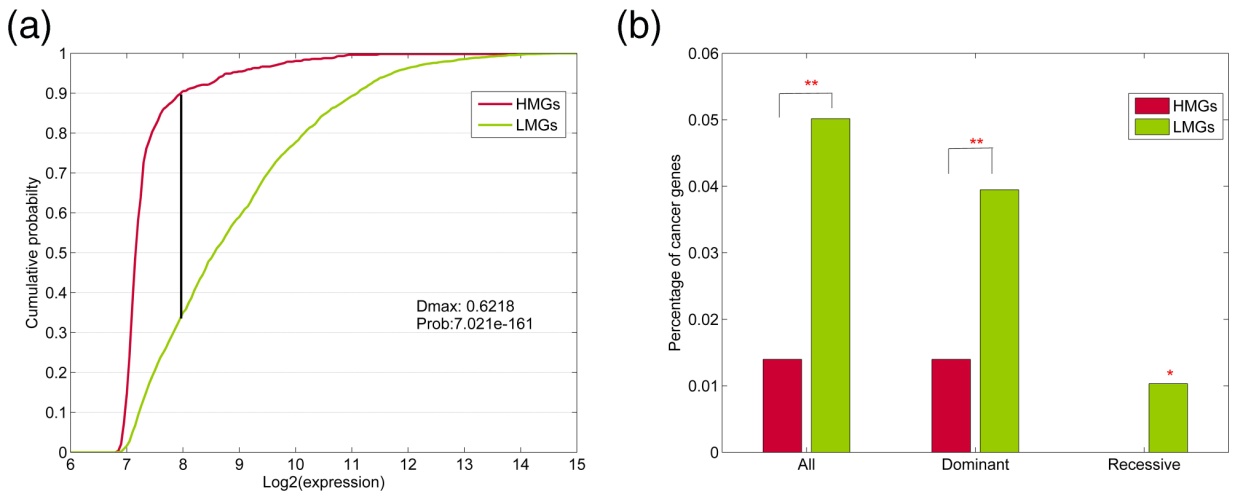


**Figure S9. LMGs and HMGs are significantly different in expression pattern and functions.** (a) The cumulative distribution functions of gene expression for LMGs (green) and HMGs (red). (b) Comparison of the percentage of cancer genes. Cancer genes are further divided into dominant and recessive cancer genes according the annotations of cancer gene census.

**Functional complementation between methylation and microRNA regulation**

Unexpectedly, when comparing the miRNA regulation pattern of LMGs and HMGs, we found LMGs tend to be regulated by miRNAs (Figure S10a, p-values<0.001, Fisher’s exact test). About 91.92% of LMGs are predicted to be miRNA target gene set, which is about 1.32 fold to that of HMGs (p-value<1.0e-32, Fisher’s exact test). We found the trend is clearer in the “experiment validated target set” than in the “predicted target set” (the ratio is about 3.73 times, p-value=3.814e-13, Fisher’s exact test), suggesting that the false positive information incorporated in the “predicted target set” leads to a lower fold. Moreover, the genes in LMG class regulated by miRNAs tend to have more miRNA regulations than HMGs, implying more miRNA regulatory complexity of these genes (Figure S10b, p-value=1.773e-50, Kolmogorov-Smirnov test).


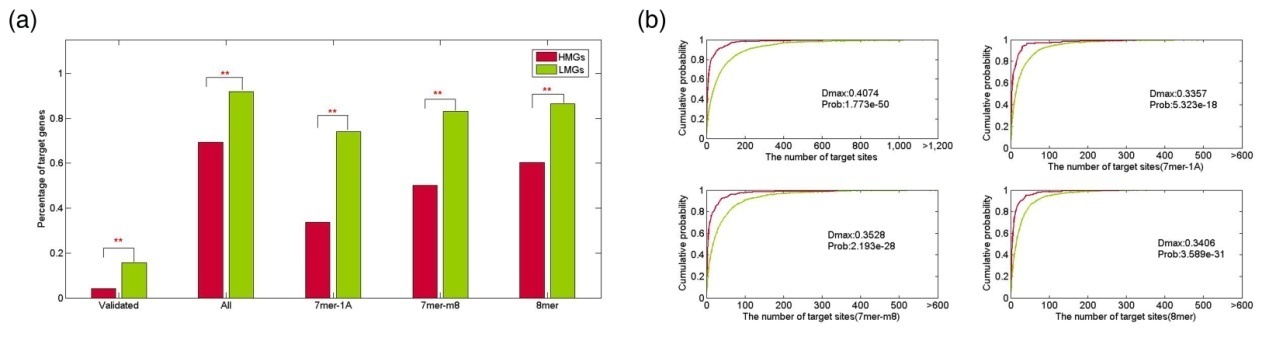


**Figure S10. LMGs and HMGs are significantly different in miRNA regulations.** (a) Comparison of the percentage of miRNA targets. The experimental validated target genes are retrieved from four manually curated databases while the predicted miRNA targets are collected from TargetScan and further divided into three types of targets. (b) The cumulative distribution functions of the number of miRNAs that regulate LMGs (green) or HMGs (red). The maximum distance between these two distributions and the probabilities are computed by the Kolmogorov-Smirnov (K-S) test.

**References**

1. Bossi A, Lehner B (2009) Tissue specificity and the human protein interaction network. Mol Syst Biol 5: 260.

2. Zhang QC, Petrey D, Deng L, Qiang L, Shi Y, et al. (2012) Structure-based prediction of protein-protein interactions on a genome-wide scale. Nature 490: 556-560.
