# Supplementary material for: Characterizing Genes with Distinct Methylation Patterns in the Context of Protein-Protein Interaction Network: Application to Human Brain Tissues
Source: PLoS One. 2013 Jun 12;8(6):e65871. doi: 10.1371/journal.pone.0065871 (PMC3680465; doi:10.1371/journal.pone.0065871)
Supplement: Text S2 — The organizational principles of genes with distinct methylation patterns are robust to different thresholds. (DOC) [file pone.0065871.s003.doc]

**Text S2. The organizational principles of genes with distinct methylation patterns are robust to different thresholds**

In order to explore if the organizational principles are robust to the threshold we used to define the LMGs and HMGs, we reanalyzed the results described in the text. We also specially focused on the two classes of genes: the first gene class which we termed as LMGs (low methylated genes), consists of genes with beta values less than 0.3 and CpG ratio greater than 0.7; and the other gene class includes genes with beta values greater than 0.7 and CpG ratio less than 0.3, named as HMGs (high methylated genes). Finally, we allocated 3,122 genes to the LMG class and 641 genes to the HMG class.

**LMGs are located in the central of the PPIN**

As discussed above, DNA methylation levels and the topological features have an inverse association. Next, we analyzed the differences in topological features of these two gene groups. A summary of these analysis results is listed in Table S1. As a result, LMGs tend to interact with more genes than HMGs and have a higher betweenness centrality. The average degree of LMGs is 9.718, which is significantly higher than that of HMGs, even significantly higher than the average degree of the whole PPIN. Moreover, the average betweenness of LMGs is more than twice to that of HMGs. These results indicate that many of the LMGs are network hubs and bottlenecks, whose values are ranked top 10% of the whole gene list; moreover, we found the LMGs are indeed overrepresented in the top genes with high number of interactions (hubs) but the HMGs are underrepresented (Figure S1a).

## Table S1 - Comparisons of topological features of LMGs and HMGs.

|  | HPRD | | LMGs | | HMGs | | Rank sum test |
| --- | --- | --- | --- | --- | --- | --- | --- |
|  | Mean | Std | Mean | Std | Mean | Std | p-values |
| Degree | 7.945 | 14.583 | 9.718 | 16.617 | 5.987 | 10.832 | 7.568e-10 |
| Betweenness (*104) | 2.914 | 12.221 | 3.810 | 13.832 | 1.860 | 8.812 | 1.930e-7 |
| Closeness | 0.241 | 0.031 | 0.247 | 0.031 | 0.232 | 0.030 | 5.232e-23 |

Next, we used an *in silico* strategy that simulated the effect of specifically removing (attacking) genes in the PPIN on the characteristic path length of the main component of the network. Actually, separately removing the LMGs and HMGs from the original network has distinct effects on the overall network integrity. Moreover, successive attacks against LMGs starting from the most connected genes have a more deleterious effect on the network integrity than the removal of random proteins (Figure S1b). Conversely, removal of HMGs does not affect connectivity and thus has similar deleterious effect as the removal of random genes. As the number of components and the size of maximum component measure the integrity of a network from another two aspects, we found the number of components after removal of LMGs is significantly larger than that of removal of HMGs (Figure S1c). However, the main component that remains after removing the LMGs is significantly smaller than that remaining after the removal of HMGs (Figure S1d).

Collectively, these results show that LMGs and HMGs have markedly different global properties in the protein interaction network. The LMGs tend to be hubs and bottlenecks in the network, indicating that they are located in the central of the protein interaction network, and play important roles in biological processes. In contrast, the HMGs are located in the periphery of the network. Attacking LMGs may cause a more deleterious effect on the network integrity than that of HMGs.


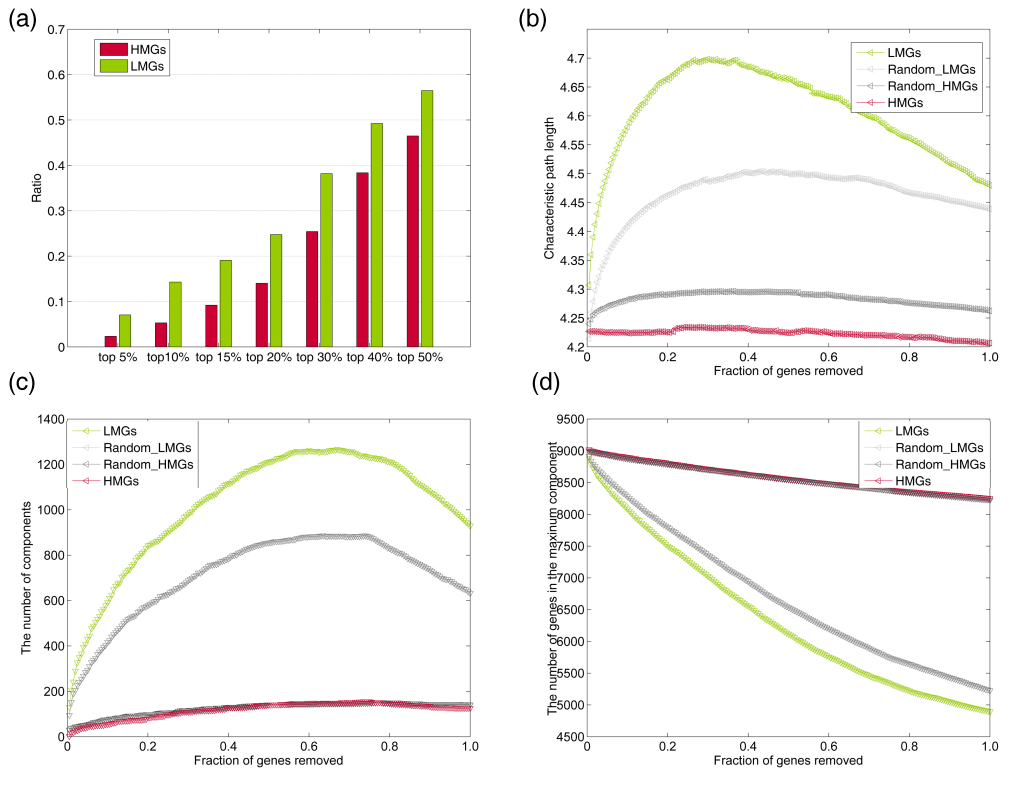


**Figure S1. LMGs are central to network topology.** (a) The percentage of LMGs and HMGs in the hubs. Genes are ranked by the degree in the PPIN and hubs are defined as the top ranked genes. (b) The effects on the characteristic path length of the network on gradual node removal. Random removal of nodes is represented by the grey lines, dark grey line represents the random removal of HMGs while light grey line represents random removal of LMGs, attacks against LMGs by the green line, attacks against HMGs by the red line. (c) The number of components remaining after removing the LMGs, HMGs and random genes. (d) The sizes of the largest remaining component after removing LMGs, HMGs and random genes.

**Modular organization of LMGs and HMGs in the protein interaction network**

We analyzed the modular and community structure of these two classes of genes. After mapping these two classes of genes to the protein interaction network, we constructed two networks of LMGs and HMGs, named as LMN and HMN. The maximum component of LMN consists of 2,491 genes, and it is much larger than expected by chance (Figure S2a, p-value<1.0e-4). Furthermore, there are 6,624 edges between genes in the maximum component, which is much denser than expected by chance (Figure S2c, p-value<1.0e-4). However, the maximum component of HMG network only has 171 genes connected by 191 edges, which is significantly smaller than random conditions (Figure S2b, d).

Next, we further used three common metrics to measure the modularity of a subnetwork (see methods). As a result, we found the LMN shows significantly higher network modularity than what would be expected in random conditions (Table S2). The characteristic path lengths between the LMGs are significantly shorter (Figure 4a, 4.028 on average, p-value<0.001), implying that the LMGs are closer to each other. In addition, the LMN also exhibits significantly higher in-degree ratio and density. However, the characteristic path lengths between HMGs are significantly longer than random conditions (Figure S3a). The average ratio of in-degree of HMGs is only 0.071, implying that the proteins with high methylation levels may not always form a module. Conversely, the HMN1 exhibits significant modular features (Table S2). These analyses indicated that LMGs express their function in a modular pattern, while although genes with higher methylation levels might not form a network module themselves, they are with the aid of their interacting partners to show significantly higher modularity.


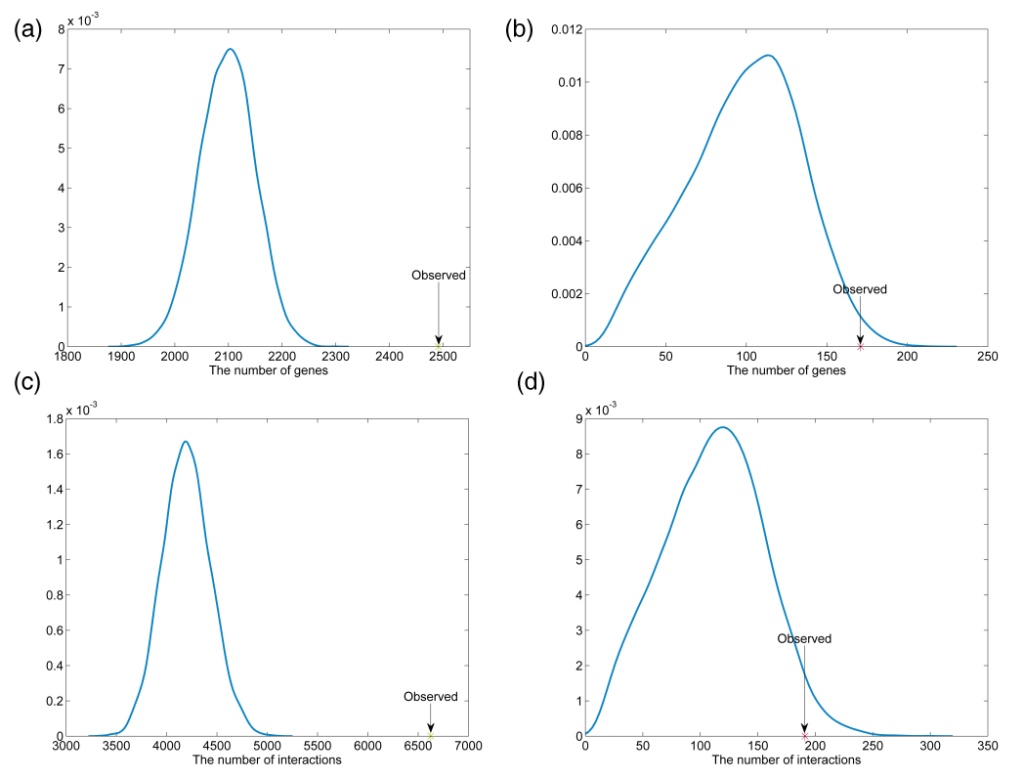


**Figure S2. The LMG and HMG networks.** (a) The number of vertexes of LMN is significantly larger than that of random networks (p-value<1.0e-4). (b) The number of vertexes of HMN is similar to random networks. (c) The number of edges of LMN is significantly larger than that of random networks (p-value<1.0e-4). (d) The number of edges of HMN is similar to random networks.

## Table S2 - Summary of modular properties of LMGs and HMGs.

|  | HPRD | LMG network | | H0 network | | H1 network | |
| --- | --- | --- | --- | --- | --- | --- | --- |
|  | Mean | Mean | p-value | Mean | p-value | Mean | p-value |
| In-degree ratio | N/A | 0.2816 | <0.001 | 0.071 | <0.001 | 0.448 | <0.001 |
| Density | 8.77e-4 | 0.002 | <0.001 | 0.001 | 0.007 | 0.004 | <0.001 |
| Characteristic path length | 4.227 | 4.0284 | <0.001 | 4.443 | 1 | 3.814 | <0.001 |

Additionally, to estimate if the LMGs still tend to be within the same densely connected modules detected in the original PPIN, we used the CFinder tool to identify modules from the whole PPIN. We defined genes from the LMGs or HMGs playing important roles in a module only if half or more members of the module are the LMGs or HMGs. And then we counted the number of modules for the LMGs and HMGs respectively. As shown in the Figure S3b, with the minimum number of genes in modules (k) increases, there is a sharp decrease in the number of HMG-involved modules, indicating that the HMGs do not tend to be assembled in the same modules. In contrast, the LMGs participate in more modules even some big ones. As discussed above, the LMGs implement functions as modules and they are located in close proximity.

**
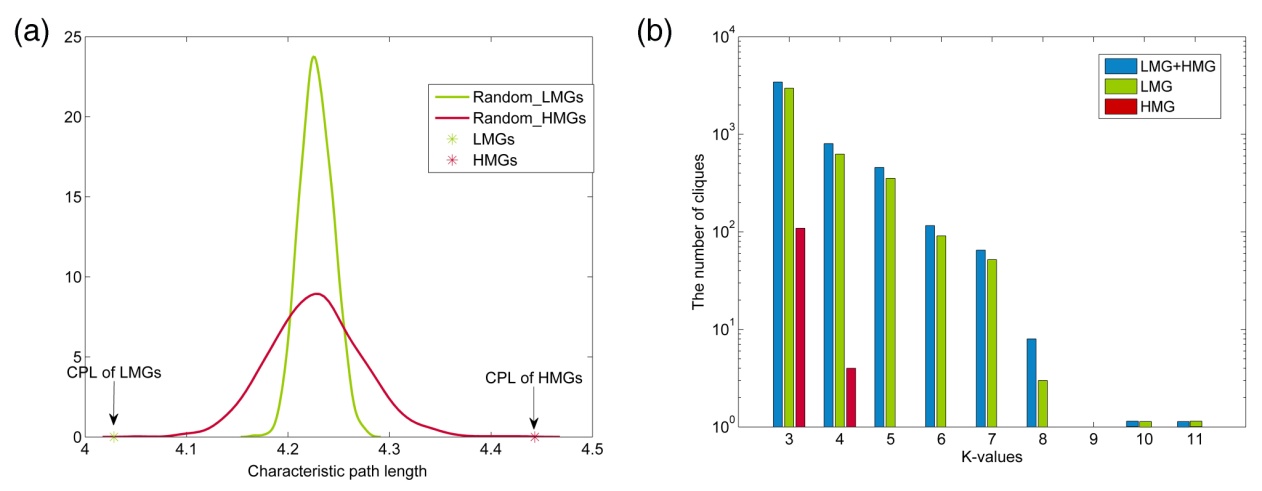
**

**Figure S3. LMGs resemble functional modules in the PPIN.** (a) Comparison of average lengths of shortest paths among LMGs, HMGs and random genes in the human protein interaction network from HPRD database. The distance between random LMGs or HMGs is fitted. (b) Number of cliques with the percentage of interesting genes larger than 0.5 at different k-values. (c)-(h) Examples of LMGs communities. Genes that are detailedly analyzed in the text are marked with red stars.

**Interaction preferences of LMGs and HMGs**

In order to understand how genes with different methylation levels assembled within the protein interaction network, here, we analyzed the interaction preferences of these two classes of genes. For this purpose, we defined interaction preference index to find out significant over-represented or under-represented interaction patterns (see details in methods). Consistent with our results in main text, there is a significantly high density of interactions among LMGs or among the HMGs, implying the communications of intra-class are enhanced (Figure S4a, p-values<0.001). However, the interaction density between LMGs and HMGs appears to be extremely low, and the interactions among LMGs and HMGs are significantly repressed (Figure S4b, p-values<0.001).


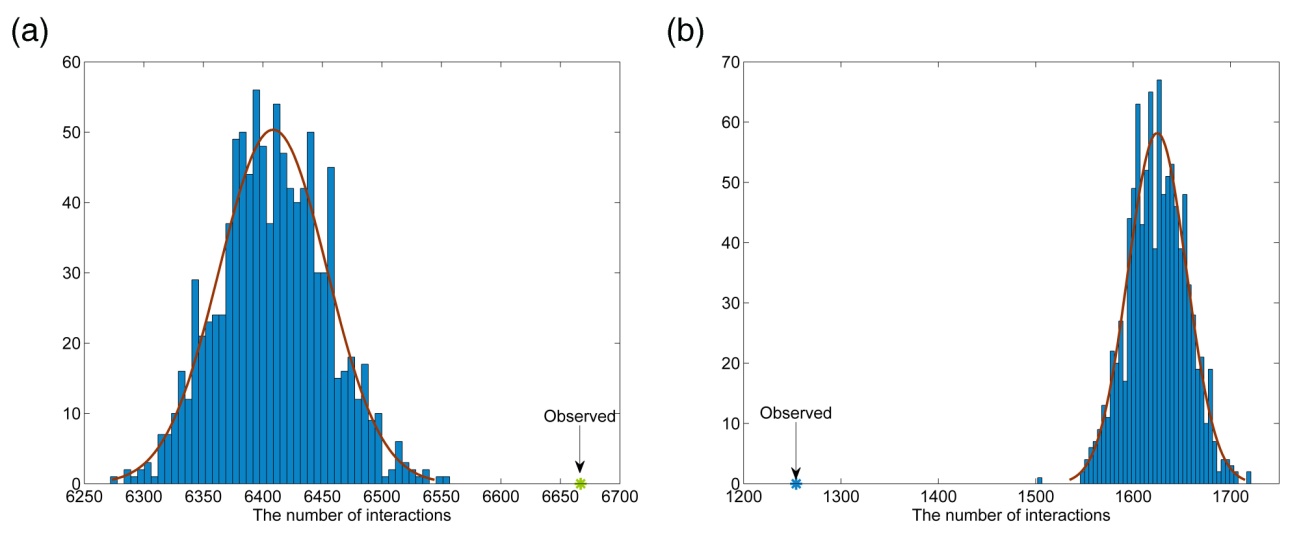


**Figure S4. Interaction preferences of LMGs and HMGs.** (a) The number of interactions within LMGs is significantly larger than that of degree-conserved random networks (p-value<0.001). (b) The number of interactions between LMGs and HMGs is significantly smaller than that of degree-conserved random networks (p-value<0.001). The procedure to generate the random networks is described in materials and methods.

**Differences in expression and functions between the LMGs and HMGs**

We found the expression patterns between LMGs and HMGs are significantly different (p-value=2.342e-134, Kolmogorov-Smirnov test), and the LMGs are enriched in high expression genes (Figure S5a), implying its key roles in brain tissues. In addition, some genes may belong to a class of genes that play specific roles in cellular systems; it is interesting to examine the DNA methylation pattern of these genes, which may provide new insights into understanding the mechanism of complex diseases. Firstly, we explored the methylation patterns of cancer genes. As expected, we found the cancer genes are significantly over-represented in the LMGs (Figure S5b, p-value=0.0019, Fisher’s exact test), indicating cancer genes tend to have low methylation levels, which is consistent with a recent study. We next compared the DNA methylation patterns between two major classes of cancer genes: dominant and recessive cancer genes. After excluding four genes with ambiguous classification in the database, among the 470 cancer genes, there were 365 dominant cancer genes and 105 recessive cancer genes. Interestingly, the dominant cancer genes are slightly overrepresented in the LMG class (p-value=0.0103, Fisher’s exact test), while recessive cancer genes are slightly over-presented in the HMG class, indicating that recessive genes tend to avoid methylation in normal tissues. When we compared the distribution of essential genes in two classes of genes with different methylation levels, we found essential genes are significantly overrepresented in LMGs than HMGs, indicating essential genes also tend to have low methylation levels in normal tissues (p-value=3.42e-7, Fisher’s exact test). Finally, we found the aging genes are also over-represented in the LMG class (p-value=0.0013, Fisher’s exact test), indicating aging genes tend to have lower methylation levels.


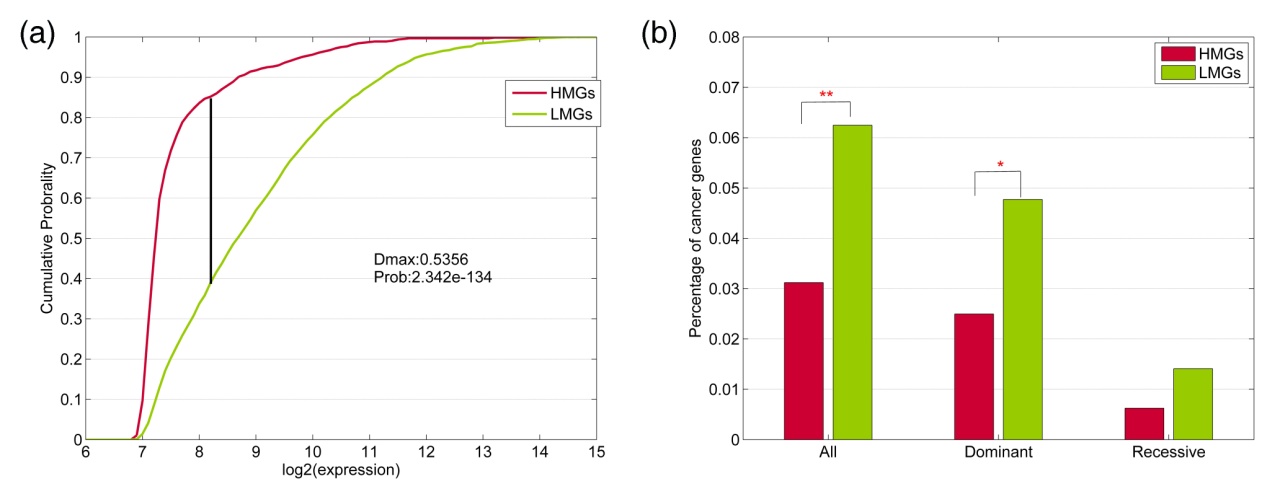


**Figure S5. LMGs and HMGs are significantly different in expression pattern and functions.** (a) The cumulative distribution functions of gene expression for LMGs (green) and HMGs (red). (b) Comparison of the percentage of cancer genes. Cancer genes are further divided into dominant and recessive cancer genes according the annotations of cancer gene census.

**Functional complementation between methylation and microRNA regulation**

Unexpectedly, when comparing the miRNA regulation pattern of LMGs and HMGs, we found LMGs tend to be regulated by miRNAs (Figure S6a, p-values<0.001, Fisher’s exact test). About 92.70% of LMGs are predicted to be miRNA target gene set, which is about 1.25 fold to that of HMGs (p-value<1.0e-32, Fisher’s exact test). We found the trend is clearer in the “experiment validated target set” than in the “predicted target set” (the ratio is about 2.57 times, p-value=3.001e-12, Fisher’s exact test), suggesting that the false positive information incorporated in the “predicted target set” leads to a lower fold. Moreover, the genes in LMG class regulated by miRNAs tend to have more miRNA target sites than HMGs, implying more miRNA regulatory complexity of these genes (Figure S6b, p-value=2.379e-41, Kolmogorov-Smirnov test).


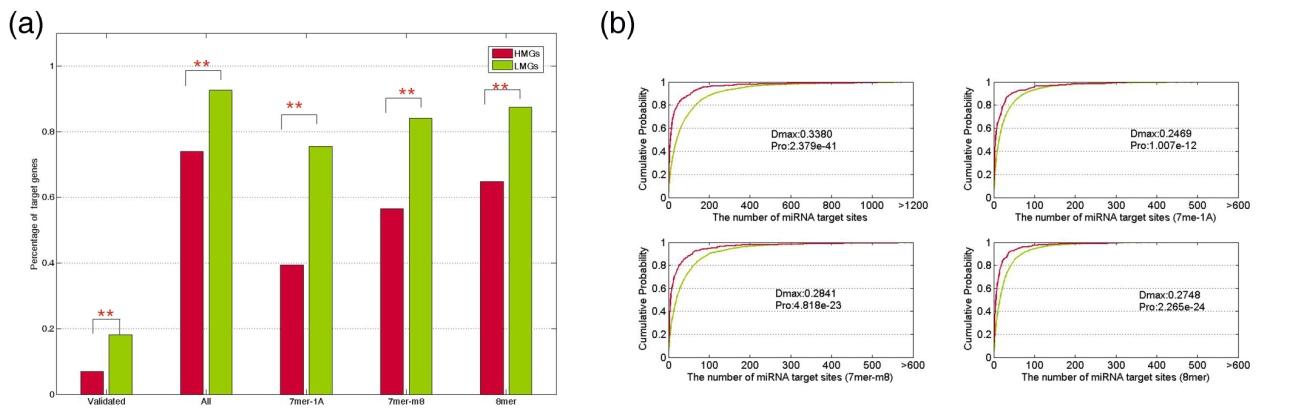


**Figure S6. LMGs and HMGs are significantly different in miRNA regulations.** (a) Comparison of the percentage of miRNA targets. The experimental validated target genes are retrieved from four manually curated databases while the predicted miRNA targets are collected from TargetScan and further divided into three types of targets. (b) The cumulative distribution functions of the number of miRNA target sites that in LMGs (green) or HMGs (red). The maximum distance between these two distributions and the probabilities are computed by the Kolmogorov-Smirnov (K-S) test.
