# Supplementary material for: Characterizing Genes with Distinct Methylation Patterns in the Context of Protein-Protein Interaction Network: Application to Human Brain Tissues
Source: PLoS One. 2013 Jun 12;8(6):e65871. doi: 10.1371/journal.pone.0065871 (PMC3680465; doi:10.1371/journal.pone.0065871)
Supplement: Text S3 — The topological features of intermediate methylated genes. (DOC) [file pone.0065871.s004.doc]

**Text S3. The topological features of intermediate methylated genes**

In the main text, we only focused on the LMGs and HMGs, it might be interesting as well, where the intermediate class stays. Using the 0.2 and 0.8 as threshold, we obtained 2,018 genes with CpG ratio and beta values are within 0.2-0.8, which are defined as intermediated methylated genes (IMGs). When comparing the topological features of the IMGs with LMGs, HMGs and the whole PPIN, we found the IMGs have the intermediate degree, betweenness and closeness. The topological features of these three group genes are listed in Table S1.

## Table 1 - Comparisons of topological features of LMGs, IMGs and HMGs.

|  | HPRD | | LMGs | | IMGs | | HMGs | |
| --- | --- | --- | --- | --- | --- | --- | --- | --- |
|  | Mean | Std | Mean | Std | Mean | Std | Mean | Std |
| Degree | 7.945 | 14.583 | 9.969 | 17.112 | 7.533 | 12.628 | 5.450 | 6.946 |
| Betweenness (*10^4^) | 2.914 | 12.221 | 3.897 | 14.671 | 2.659 | 9.010 | 1.303 | 2.823 |
| Closeness | 0.241 | 0.031 | 0.248 | 0.030 | 0.239 | 0.032 | 0.231 | 0.030 |

In addition, we found the topological features of LMGs are significantly larger than randomly selected genes while the HMGs’ topological features are significantly smaller than randomly selected genes. However, the topological features of IMGs are similar to randomly selected genes (Figure S1). Next, we explored the ratio of hub in these three groups of genes. As a result, we found the LMGs are overrepresented in hubs, while HMGs are underrepresented in hubs and the IMGs are similar to the whole PPIN (Figure S2).


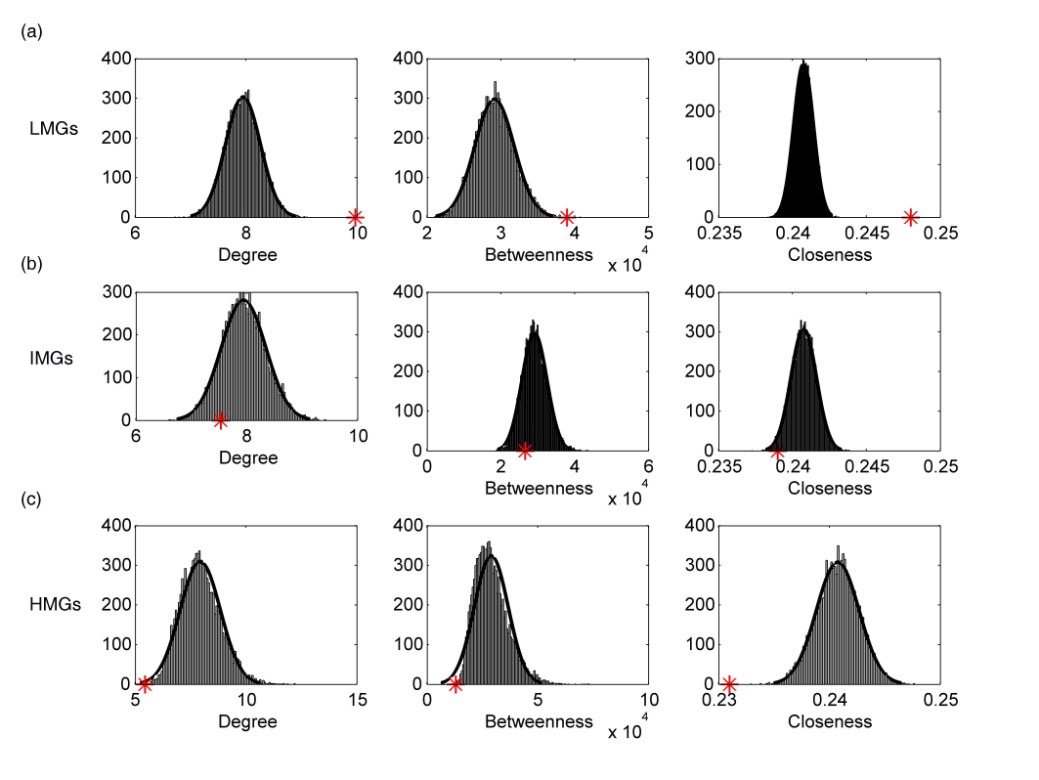


**Figure S1. The topological features of LMGs, IMGs and HMGs compared to randomly selected genes.** (a) The degree, betweenness and closeness of LMGs. (b) The degree, betweenness and closeness of IMGs. (c) The degree, betweenness and closeness of HMGs.


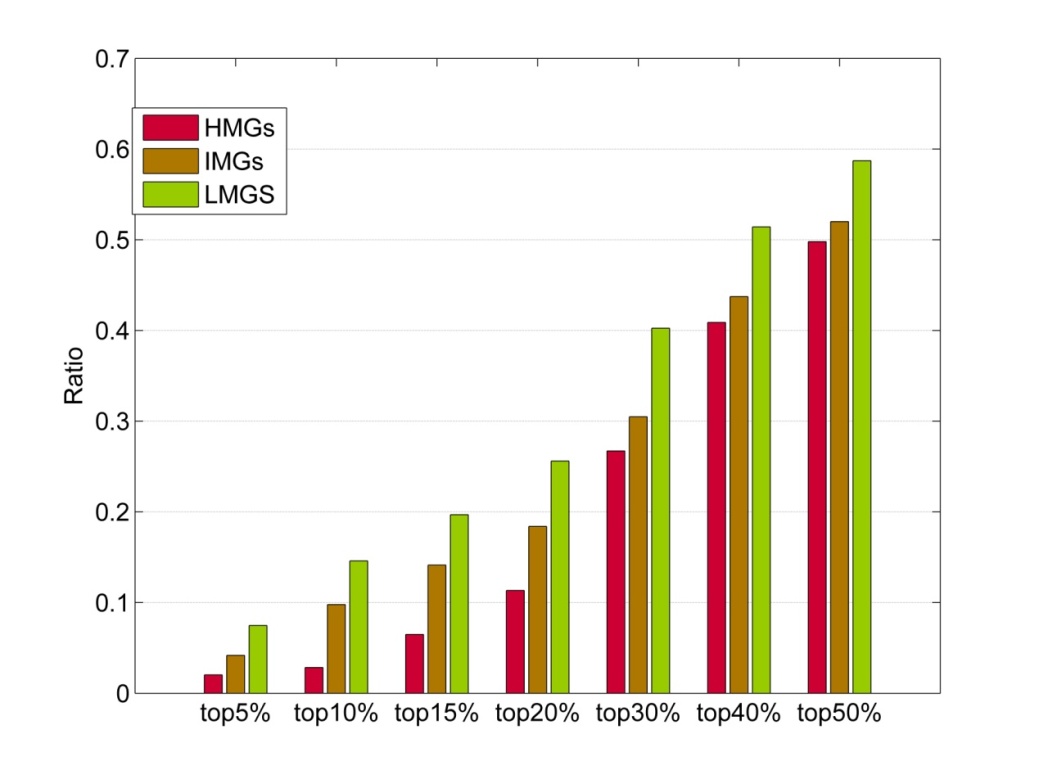


**Figure S2. The ratio of hubs in three groups of genes.**
